# Phenanthroindolizidine Alkaloids Isolated from *Tylophora ovata* as Potent Inhibitors of Inflammation, Spheroid Growth, and Invasion of Triple-Negative Breast Cancer

**DOI:** 10.3390/ijms231810319

**Published:** 2022-09-07

**Authors:** Irene Reimche, Haiqian Yu, Ni Putu Ariantari, Zhen Liu, Kay Merkens, Stella Rotfuß, Karin Peter, Ute Jungwirth, Nadine Bauer, Friedemann Kiefer, Jörg-Martin Neudörfl, Hans-Günther Schmalz, Peter Proksch, Nicole Teusch

**Affiliations:** 1 Department of Biomedical Sciences, Institute of Health Research and Education, University of Osnabrück, 49090 Osnabrück, Germany; 2 Institute of Pharmaceutical Biology and Biotechnology, Heinrich Heine University, 40225 Düsseldorf, Germany; 3 Department of Pharmacy, Faculty of Mathematics and Natural Sciences, Udayana University, Bali 80361, Indonesia; 4 Department of Chemistry, University of Cologne, 50923 Cologne, Germany; 5 Department of Life Sciences, Centre for Therapeutic Innovation, University of Bath, Bath BA2 7AY, UK; 6 European Institute of Molecular Imaging, University of Münster, 48149 Münster, Germany; 7 Max Planck Institute for Molecular Biomedicine, 48149 Münster, Germany

**Keywords:** triple-negative breast cancer (TNBC), phenanthroindolizidine alkaloids, *O*-methyltylophorinidine, nuclear factor kappa B (NFκB), hypoxia-inducible factor (HIF), tumor microenvironment (TME), 3D spheroid, cancer-associated fibroblast (CAF)

## Abstract

Triple-negative breast cancer (TNBC), representing the most aggressive form of breast cancer with currently no targeted therapy available, is characterized by an inflammatory and hypoxic tumor microenvironment. To date, a broad spectrum of anti-tumor activities has been reported for phenanthroindolizidine alkaloids (PAs), however, their mode of action in TNBC remains elusive. Thus, we investigated six naturally occurring PAs extracted from the plant *Tylophora ovata*: *O*-methyltylophorinidine (**1**) and its five derivatives tylophorinidine (**2**), tylophoridicine E (**3**), 2-demethoxytylophorine (**4**), tylophoridicine D (**5**), and anhydrodehydrotylophorinidine (**6**). In comparison to natural (**1**) and for more-in depth studies, we also utilized a sample of synthetic *O*-methyltylophorinidine (**1s**). Our results indicate a remarkably effective blockade of nuclear factor kappa B (NFκB) within 2 h for compounds (**1**) and (**1s**) (IC_50_ = 17.1 ± 2.0 nM and 3.3 ± 0.2 nM) that is different from its effect on cell viability within 24 h (IC_50_ = 13.6 ± 0.4 nM and 4.2 ± 1 nM). Furthermore, NFκB inhibition data for the additional five analogues indicate a structure–activity relationship (SAR). Mechanistically, NFκB is significantly blocked through the stabilization of its inhibitor protein kappa B alpha (IκBα) under normoxic as well as hypoxic conditions. To better mimic the TNBC microenvironment in vitro, we established a 3D co-culture by combining the human TNBC cell line MDA-MB-231 with primary murine cancer-associated fibroblasts (CAF) and type I collagen. Compound (**1**) demonstrates superiority against the therapeutic gold standard paclitaxel by diminishing spheroid growth by 40% at 100 nM. The anti-proliferative effect of (**1s**) is distinct from paclitaxel in that it arrests the cell cycle at the G0/G1 state, thereby mediating a time-dependent delay in cell cycle progression. Furthermore, (**1s**) inhibited invasion of TNBC monoculture spheroids into a matrigel^®^-based environment at 10 nM. In conclusion, PAs serve as promising agents with presumably multiple target sites to combat inflammatory and hypoxia-driven cancer, such as TNBC, with a different mode of action than the currently applied chemotherapeutic drugs.

## 1. Introduction

Worldwide, breast cancer (BC) is the leading type of cancer among women, with an estimated 2.3 million new BC cases and 685,000 BC-related deaths in 2020 [1]. Subtype categorization is based on the expression of estrogen receptor (ER), progesterone receptor (PR), or human epidermal growth factor receptor 2 (EGFR2/HER2). Triple-negative breast cancer (TNBC), apart from the principle of genetic heterogeneity, lacks significant expression of these receptors (ER-/PR-/HER2-) and accounts for 10–20% of all BC cases, mostly among younger women [2,3], and a disproportionate 83% of deaths in comparison with other hormone receptor-positive or HER2-positive subtypes of BC irrespective of age and race [4]. TNBC is characterized by a high rate of proliferation, metastasis, and shorter overall survival due to recurrence after chemotherapy based on taxanes (e.g., paclitaxel), anthracyclines (e.g., doxorubicin), or platinum-based treatment regimens [5,6,7,8]. Thus, the lack of targeted therapy options for TNBC emphasizes the urgent need for identifying novel treatment regimens. 

Compared to other BC subtypes, the nuclear factor kappa B (NFκB) is overexpressed [9] and highly activated in TNBC [10], which correlates with poor clinical outcome [9]. NFκB is a main regulator in inflammation and drives tumor aggressiveness in breast and other cancer types [11,12,13,14,15,16]. In mammalians, the NFκB family comprises five functionally conserved transcription factors: p50 (processed from p105), p52 (processed from p100), RelA (p65), RelB, and c-Rel with p50/p65 representing the predominant dimer in ER-BC types [17]. P50/p65 is retained in the cytoplasm, when engaged to its inhibitor protein kappa B alpha (IκBα). Various NFκB activating stimuli, e.g., paclitaxel or the tumor necrosis factor α (TNFα), trigger the complex of IκB kinase (IKK)α/IKKβ/IKKϒ, which phosphorylates the IκBα/NFκB complex. Subsequently, IκBα is degraded via the proteasome and the p50/p65 dimer is released to translocate to the nucleus [18,19]. Among over 150 target genes, pro-inflammatory cytokines interleukin (IL) 6 and IL-8 maintain a positive feedback loop via NFκB to orchestrate tumor growth, metastasis, and drug resistance in an autocrine manner [20,21]. In particular, migration, as a crucial step in metastasis, is regulated by NFκB through the activation of transcription factors involved in the endothelial-to-mesenchymal transition (EMT) [22]. Moreover, inhibiting NFκB signaling is sufficient to suppress cancer cell migration and invasion [23] and to reverse paclitaxel resistance [19]. Thus, NFκB represents a promising target in TNBC.

Tumor tissue comprising rapidly proliferating cancer cells is characterized by poor vascularization, and compared to other breast cancer subtypes TNBC tissue is characterized by low oxygen levels [24]. Hypoxia is an important feature of the tumor microenvironment (TME) of TNBC and is associated with aggressiveness, invasiveness, and resistance to therapy [25]. In this context, poor clinical outcome is linked to a high activity of the transcription factor hypoxia-inducible factor 1alpha (HIF-1α), which is stabilized under hypoxia, thereby mediating cell survival [26,27]. The HIF transcription factor family comprises oxygen-sensing α-subunits (HIF-1α, HIF-2α, HIF-3α) and oxygen-insensitive β-subunits (e.g., HIF-1β), both dimerizing to form heterodimeric HIF-transcription complexes under hypoxic conditions, stabilizing HIF-driven gene expression. During normoxia, oxygen-sensing HIF-1α is hydroxylated and degraded via the proteasome, whereby under hypoxic conditions, hydroxylation is impaired due to oxygen shortage, resulting in HIF-1α stabilization, its nuclear translocation, and subsequent transcription of target genes related to angiogenesis, glycolysis, migration, and tumor progression, also via crosstalk with other pathways, such as NFκB [28]. Hypoxia is known to enhance NFκB activity [29], while, in turn, NFκB transcriptionally regulates HIF [28]. Both pathways, NFκB as well as HIF, are described as key drivers for cancer stem cells through mediating EMT [30]. Indeed, cancer stem cells are enriched in TNBC [31] and correlate with worse clinical outcome [32] due to their high capacity for self-renewal, enhanced metastasis, and chemotherapy resistance resulting in tumor relapse that is a major cause of therapy failure [30]. Paclitaxel is reported to enhance HIF and NFκB activity and, inversely, blocking both pathways could reduce chemotherapy resistance [19,24,33]. Thus, targeting NFκB and HIF may improve a patient’s outcome by targeting key processes for drug resistance. 

The TNBC TME is an ensemble of endothelial cells, immune cells, adipocytes, and fibroblasts, in addition to the soluble factors released from all the cellular components (including cancer cells) [34,35,36]. Paracrine signaling cancer cells recruit non-cancerous stromal cells to the tumor site to create an inflammatory environment that drives and maintains tumor progression [37]. Stromal cells are enriched in the TNBC TME [38], and pro-inflammatory cytokines activate fibroblasts to so-called cancer-associated fibroblasts (CAFs), which are the predominant stromal cell population [37]. CAFs recruit immunosuppressive cells [39] and are correlated with poor clinical outcome in TNBC patients [40] through mediating tumor growth, metastasis, and drug resistance [41]. CAFs display a high activation status of NFκB, which is crucial to maintaining its pro-tumorigenic features, and targeting NFκB reverses the active fibroblast state [42]. CAFs support tumor invasion and metastasis by enhanced secretion of extracellular matrix (ECM) components, mainly collagen, and proteases for ECM degradation [43]. Enhanced collagen type I acts as a physical barrier and correlates with reduced drug response [32,44]. Furthermore, a major role in tumor progression is attributed to the CAF-derived cytokines and growth factors that engage NFκB and HIF pathways [30,45], and correlate with cancer relapse and poor prognosis [32]. In a paracrine manner, diverse key pathways that are involved in cancer progression converge in NFκB signaling [46], which is a key pathway in mediating drug resistance [19]. Disrupting the interplay of stromal cells and TNBC through blocking NFκB suppresses paracrine signaling of cytokines IL-6 and IL-8 [23,47] and reduces tumor progression [14,42]. Thus, interfering with key signaling pathways in the TME, e.g., NFκB, that promote CAF activity and tumor progression, might represent a promising approach to combating TNBC.

In recent decades natural products have gained interest in the cancer research field due to their broad bioactivity [48]. More than half of the currently used anti-cancer drugs are either natural products or natural product derivatives, and several important chemotherapeutic drugs are derived from plants, such as taxanes and their analogues, underlining the important role of plant-derived natural products in anti-cancer drug discovery [49]. Anti-cancer compounds of the class of phenanthroindolizidine alkaloids (PAs) originate from the plant family Apocynaceae, including members of the genus *Tylophora*, which are endogenous to (sub-)tropical Africa, Asia, and Australia [50,51]. Naturally occurring PAs and their synthetic analogues show a broad range of action including anti-asthmatic [52], antiparasitic [53], antibacterial [54], antifungal [55], antiviral [56], anti-inflammatory [57], antiangiogenic [58,59], and anti-proliferative properties in vitro and in vivo, e.g., in a hepatocellular carcinoma xenograft model [60]. The anti-tumor mechanism has been described to depend on the blockade of cell signaling, e.g., NFκB in HepG2 [61] or HIF in T47D [62], and also the suppression of DNA replication and protein synthesis [63,64]. Regarding breast cancer, tylophorine acts as an anti-proliferative in the TNBC cell line MDA-MB-231 and the luminal BC cell line MCF7 as well as T47D [65,66,67], whereas in T47D tylophorine was sufficient to enhance drug sensitivity to doxorubicin [68]. However, so far, no studies have focused on TNBC to investigate the pharmacological mode of action, and thus we aimed to study the potential of PAs as novel candidates to combat inflammatory and hypoxia-driven cancer, such as TNBC. 

In our study we examined six naturally occurring derivatives isolated from the plant *Tylophora ovata*: *O*-methyltylophorinidine (**1**), tylophorinidine (**2**), tylophoridicine E (**3**), 2-demethoxy-tylophorine (**4**), tylophoridicine D (**5**), and anhydrodehydrotylophorinidine (**6**). Additionally, we utilized *O*-methyltylophorinidine from chemical synthesis (**1s**). Our results demonstrate a strikingly potent NFκB blockade with a structure–activity relationship (SAR) distinct from the cytotoxic potential of this compound class. The compound (**1**)/(**1s**) displayed the most potent capacity to inhibit NFκB-mediated transcription (IC_50_ = 17.1 ± 2.0 nM/3.3 ± 0.2 nM) and was used for more in-depth bioactivity studies. We analyzed NFκB inhibition through the stabilization of its inhibitor IκBα, and additionally found dose-dependent blockade of HIF-mediated transcription. NFκB inhibition by PAs was tested under cobalt (II) chloride (CoCl_2_)-simulated hypoxia. The PA-induced NFκB blockade was presumably maintained by additionally targeting HIF. Distinct to paclitaxel, the anti-proliferative effects of PAs depend on a delay in cell cycle progression through an arrest in G0/G1. To evaluate anti-proliferative potential, we further mimicked the TNBC TME with MDA-MB-231 spheroids encompassed with α-smooth muscle actin (αSMA)-positive primary murine CAFs. Three-dimensional spheroid growth was distinctly reduced at 100 nM (**1**) compared to the standard-of-care drug paclitaxel. In a 3D TNBC monoculture spheroid we observed significant blockade of migration and invasion at 10 nM (**1s**). TNBC progression regarding growth and invasion was significantly blocked by *O*-methyltylophorinidine, presumably by targeting the NFκB and HIF pathways. Thus, we suggest PAs as a class of multi-targeting compounds that may be applied to treat inflammation and hypoxia-driven cancer.

## 2. Results

### 2.1. Phenanthroindolizidine Alkaloid (PA) Library

**Figure 1:**
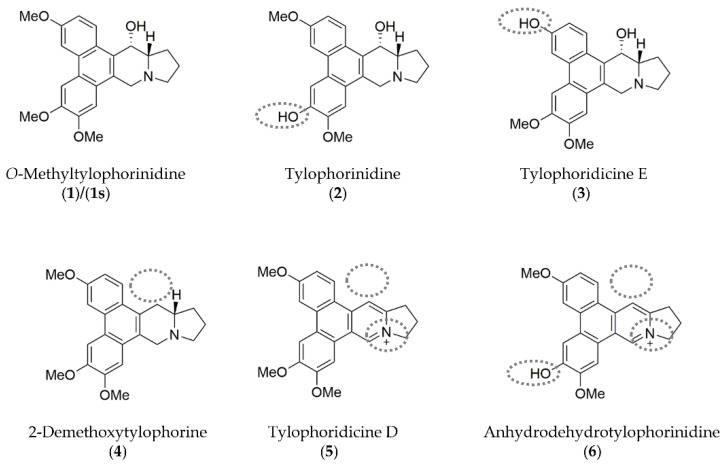
Chemical structures of the phenanthroindolizidine alkaloids (PAs) compound (**1**)/(**1s**) and its naturally occurring derivatives (compounds (**2**)–(**6**)) are shown with their distinct characteristics highlighted (grey dotted lines) compared to compound (**1**). Illustrations were drawn using ChemDraw (PerkinElmer Informatics, Hamburg, Germany; Version: 18.1.0.458).

Six phenanthroindolizidine alkaloids (PAs) were isolated from the plant *Tylophora ovata*: Compound (**1**) and its derivatives (**2**), (**3**), (**4**), (**5**), and (**6**). Additionally, due to limited amounts of the plant-derived products, *O*-methyltylophorinidine was subjected to a total synthesis, hereinafter referred to as compound (**1s**). Chemical structures and distinct characteristics are summarized in Figure 1. In parallel, we evaluated the bioactivity of the synthetically prepared (**1s**), which shares the same molecule structure with (**1**) (Figure S1). 

The core structure of all the molecules, which is based on the scaffold of compound (**1**)/(**1s**) is composed of the tricyclic phenanthrene ring fused to the bicyclic indolizidine ring. Characteristically, an α-hydroxy group at the indolizidine ring is found in compounds (**1**), (**2**), and (**3**). In comparison to (**1**), demethylation of one of the methoxy groups at the phenanthrene ring is found in (**2**), (**3**), and (**6**). Unlike the other compounds, the nitrogen at the indolizidine ring in (**5**) and (**6**) is positively charged. 

### 2.2. Phenanthroindolizidine Alkaloids (PAs) Are Potent NFκB-Inhibitors in TNBC

**Figure 2:**
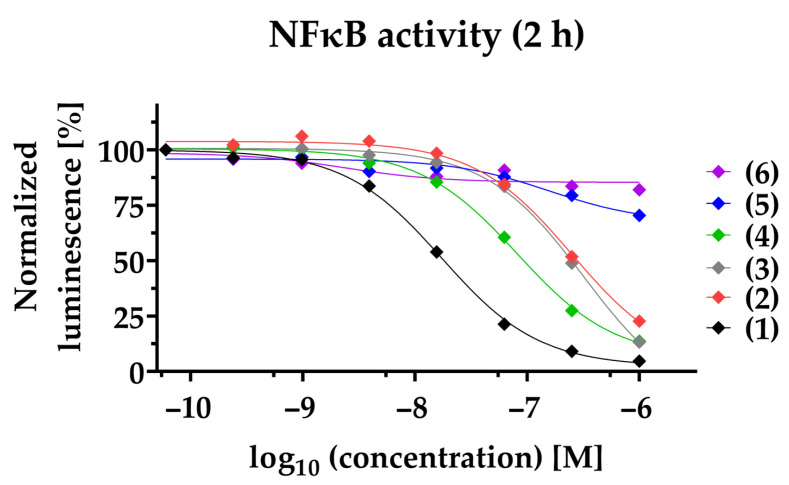
PAs inhibit NFκB-dependent gene transcription. The NFκB-dependent luciferase activity was quantified 2 h post-stimulation of NFκB-MDA-MB-231-nanoluc cells. Compounds (**1**)–(**6**) were applied at a concentration ranging from 1000 nM to 0.06 nM before being stimulated for 2 h with 20 ng/mL TNFα. In each individual experiment, the NFκB-dependent luminescence was normalized to the lowest concentration applied (100%). Each data point represents the mean of at least three independent experiments.

To study the anti-inflammatory potential of PAs in TNBC, we used a NFκB-dependent luciferase reporter cell line. Decreased NFκB transcriptional activity is observed for compounds (**1**)–(**4**) in a time- and dose-dependent manner. Compound (**1**) exhibits efficacious NFκB suppression within 2 h with a half-maximal inhibitory concentration (IC_50_) of 17.1 ± 2.0 nM, and within 24 h with a 5-fold decreased IC_50_ of 3.7 ± 1 nM (Figure 2, Table 1). Likewise, (**2**), (**3**), and (**4**) suppressed NFκB within 2 h with an IC_50_ of 211.8 ± 69.9 nM, 284.9 ± 60.4 nM, and 83.0 ± 14.7 nM, respectively. After 24 h, (**2**), (**3**), and (**4**) inhibit NFκB with a 5.5-fold (IC_50_ = 38.2 ± 14.2 nM), 2.5-fold (IC_50_ = 114.5 ± 17.9 nM), and 3- fold (IC_50_ = 28.3 ± 5.6 nM) decreased IC_50_, respectively. To clearly differentiate the blockade of NFκB pathway from cytotoxic effects, viability at 24 h post-treatment was determined in parallel (Table 1, Figure S2). Compared to NFκB blockade after 24 h, the reduction of cell viability for (**1**) and (**4**) was observed only at a about 4-fold higher concentration (IC_50_: 13.6 ± 0.4 nM and IC_50_: 127 ± 21.4 nM, respectively) and for (**2**) at a 2-fold higher concentration (IC_50_: 117.9 ± 35 nM). In contrast, compound (**3**) had a lower IC_50_ when comparing cell viability to NFκB inhibition. Notably, compounds (**5**) and (**6**) neither impaired NFκB activity (IC_50_ > 1000 nM), nor reduced cell viability (IC_50_ > 1000 nM)**.** When investigating the synthetic compound (**1s**) we observed comparable effects to those of the plant-derived original compound (**1**): NFκB blockade within 2 h with an IC_50_ of 3.3 ± 0.2 nM and decreased cell viability in MDA-MB-231 within 24 h with an IC_50_ of 4.2 ± 1 nM, validating a strong anti-inflammatory potential (Figure S1). The cytotoxicity of paclitaxel was observed with an IC_50_ of 37.4 ± 8.6 nM. Notably, the mode of action of PAs is distinct from that of the commercially available paclitaxel, and therefore it is not surprising that we could not detect blockade of NFκB within 2 h (IC_50_ > 1000 nM), or after 24 h (IC_50_ > 1000 nM) (Table 1).

**Figure 3:**
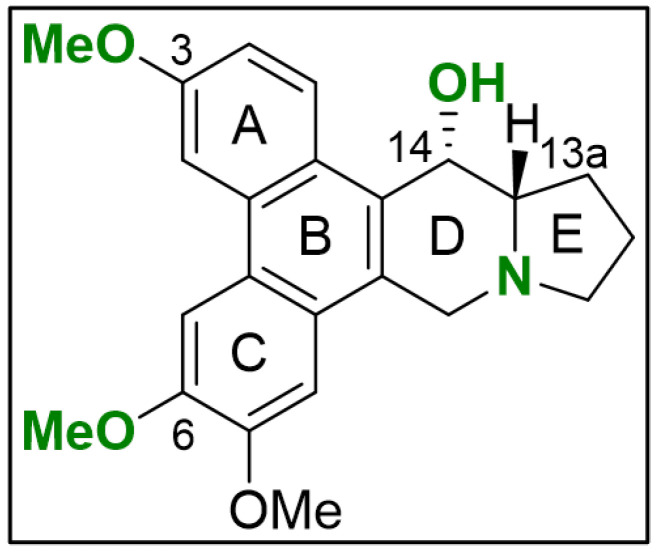
Initial SAR for PAs in TNBC. The scaffold of compounds (**1**)–(**6**) are depicted with emphasis on the chemical groups that show major influence on blocking NFκB signaling (green). The illustration was drawn using ChemDraw (PerkinElmer Informatics, Hamburg, Germany; Version: 18.1.0.458).

Evaluating the chemical structures of the PAs, we identified critical functional groups that hint at a structure–activity relationship (SAR) (Figure 3). When comparing NFκB blockade within 2 h of compound (**1**) with (**2**) and (**3**), it is evident that the loss of the methyl group at the phenanthrene decreased activity by a factor of 12 and 17, respectively. Presumably, activity is diminished through increased polarity resulting from methyl group loss in the methoxy residuals at position C-3 (ring A) in compound (**3**) and C-6 (ring C) in compound (**2**). Regarding the cytotoxic potential, the loss of the methyl group at C-3 in compound (**3**) points to a NFκB-independent anti-tumor effect, which needs further clarification. Lacking the hydroxy group at C-14 in the indolizidine ring, as found in compound (**4**), diminished NFκB blockade in comparison to compound (**1**) by a factor of 5. Especially the planar structure and the positively charged nitrogen in compound (**5**) and (**6**) significantly decrease in activity when compared to compound (**4**) representing the most critical alteration of its anti-tumor potential in TNBC.

In conclusion, PAs block NFκB-mediated transcription in TNBC in a time- and dose-dependent manner. While the compounds (**1**) and (**1s**) exhibit superior NFκB blockade, compound (**3**) may act through another mechanism. Initial SAR reveals that the anti-tumor potential is enhanced with the hydroxy group at position C-14 in the indolizidine ring, and with methoxy groups at the phenanthrene ring at positions C-3 and C-6. The planar structure and positively charged nitrogen at the indolizidine ring are dominant alterations that result in functional loss, as observed for compound (**5**) and (**6**).

### 2.3. PAs Reduce Cell Viability in the 3D TNBC Co-Culture Model

**Figure 4:**
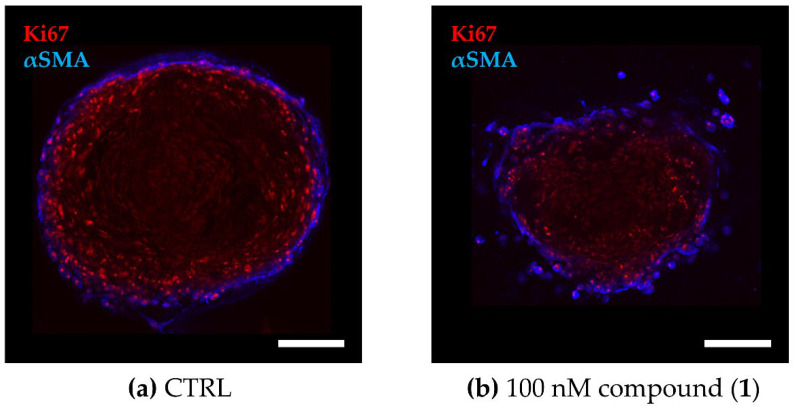
Cellular distribution within the TNBC co-culture spheroids. The 3D co-culture spheroids composed of primary murine CAFs and MDA-MB-231 cells were grown for 72 h before being treated for another 72 h. (**a**,**b**) Fluorescence images of co-culture spheroids that were (**a**) untreated (CTRL) or (**b**) treated with 100 nM of compound (**1**). Optical cross sections of the spheroids were captured after immune staining for Ki67 (red, MDA-MB-231) and αSMA (blue, CAF). Scale bar: 100 µm.

To evaluate the effect on cell viability of PAs in a more complex in vitro model that recapitulates the microenvironment better than classical 2D monocultures, we established a 3D co-culture model of the TNBC cell line MDA-MB-231 and primary murine CAFs grown in a collagen-based matrix. A distinct cellular distribution pattern within the spheroids reveals a CAF layer encompassing the tumor cell core (Figure 4), reflecting the CAF-enriched tumor stroma in TNBC [43].

The effect on cell viability of PAs was assessed in parallel in the 3D co-culture spheroids and in single cell type (MDA-MB-231 or CAF) 2D cultures (Table 1, Table 2 and Table S1). Compound (**1**) represents the most cytotoxic agent in the co-culture spheroids with an IC_50_ of 21.7 ± 2.5 nM, with only a minor increase in comparison to the 2D IC_50_. In contrast, compounds (**2**), (**3**), and (**4**) exhibit reduced cytotoxicity in the 3D co-culture model compared to 2D, presenting an at least 4-fold higher IC_50_ of 441.5 ± 70.8 nM, 476.7 ± 160.4 nM, and 517.7 ± 76.8 nM, respectively. As expected, no cytotoxic activity was observed for compounds (**5**) and (**6**) in MDA-MB-231 or in the 3D co-culture spheroids. Regarding the effect of cell viability in CAFs, the bioactivity of compounds (**1**)–(**4**) was comparable to their activities in MDA-MB-231 (Table S1).

In conclusion, PAs maintain their cytotoxicity profile in the 3D TNBC co-culture spheroids with (**1**) representing the most potent anti-tumor compound. But distinct from compound (**1**), compounds (**2**), (**3**), and (**4**) are significantly less active in the 3D co-culture model.

### 2.4. Compound (***1***) Exhibits Superior Anti-Tumor Capacity against TNBC Compared to Paclitaxel

We compared the most efficacious compound (**1**) with the chemotherapeutic agent paclitaxel regarding the cell viability and growth of co-culture spheroids. Although both compounds, (**1**) (IC_50_ = 21.7 ± 2.5 nM) and paclitaxel (IC_50_ = 43 ± 14.3 nM), maintained a dose-dependent cytotoxicity in the TNBC co-culture model, paclitaxel was not capable of inducing complete cell killing. Despite a dose-dependent response to paclitaxel, there is a cell population that is not affected under paclitaxel treatment in monolayer MDA-MB-231 as well as in 3D co-culture spheroids represented by the area under the curve (Figure S3).

**Figure 5:**
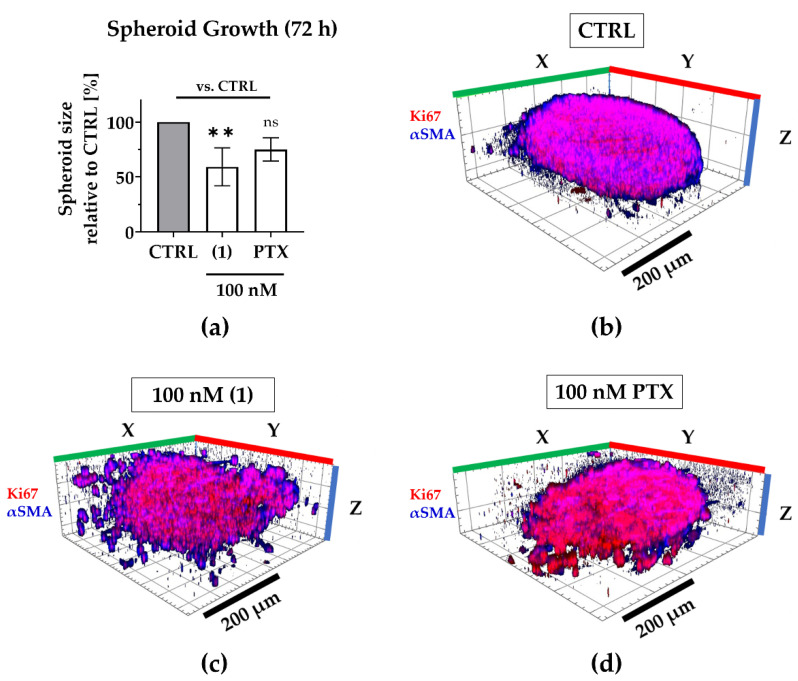
Compound (**1**) exhibits superior anti-growth effects compared to paclitaxel. The TNBC co-culture spheroids were grown for 72 h without (CTRL) or with 100 nM (**1**), 100 nM paclitaxel (PTX). (**a**) Post-treatment, the spheroid size was determined based on the spheroid area that was measured in brightfield images. The spheroid size was normalized to the mean spheroid size of CTRL (100%). Statistical significance vs. CTRL was calculated with one-way ANOVA (ns = not significant, ** *p* < 0.01). Columns represent the mean ± SD (*n* = 3). (**b**–**d**) Spheroids were immunologically stained for Ki67 (red; MDA-MB-231) and αSMA (blue; CAFs). Whole 3D spheroids were illustrated based on z-stacked fluorescence images using Zen (Zeiss, Oberkochen, Germany; blue edition, version 2.3). Scale bar: 200 µm.

Regarding 3D tumor growth inhibition, compound (**1**) significantly suppressed spheroid growth by an average of 40% compared to the untreated spheroids, while paclitaxel reduced growth only by an average of 25%, that is to say, not significantly (Figure 5). During compound treatment, the encapsulation of CAFs was maintained even though the spheroid shape was disrupted, presumably by targeting both, MDA-MB-231 as well as CAFs (compare Figure 4).

In conclusion, the natural compound (**1**) has superior anti-growth effects in TNBC co-culture spheroids compared to paclitaxel. Thus, it may serve as a novel drug candidate for treating TNBC.

### 2.5. Compound (***1s***) Blocks Invasion of TNBC Monoculture 3D Spheroids

**Figure 6:**
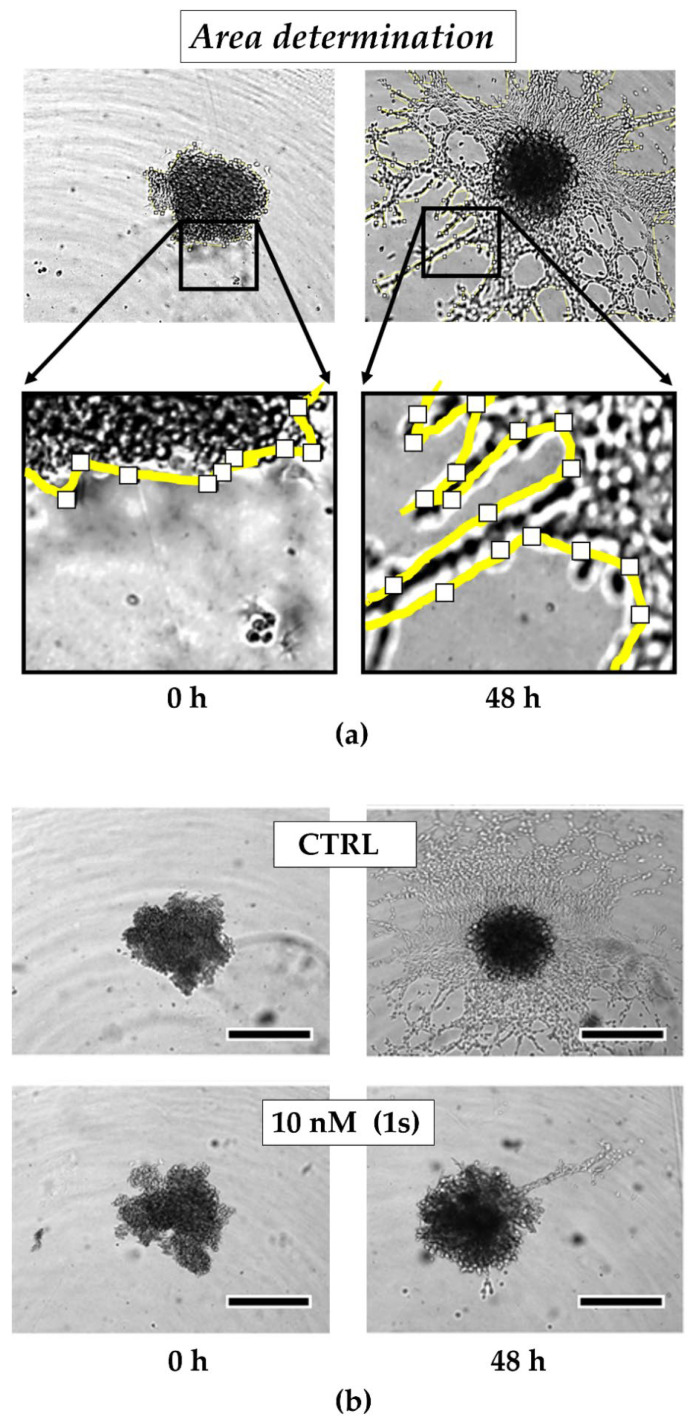

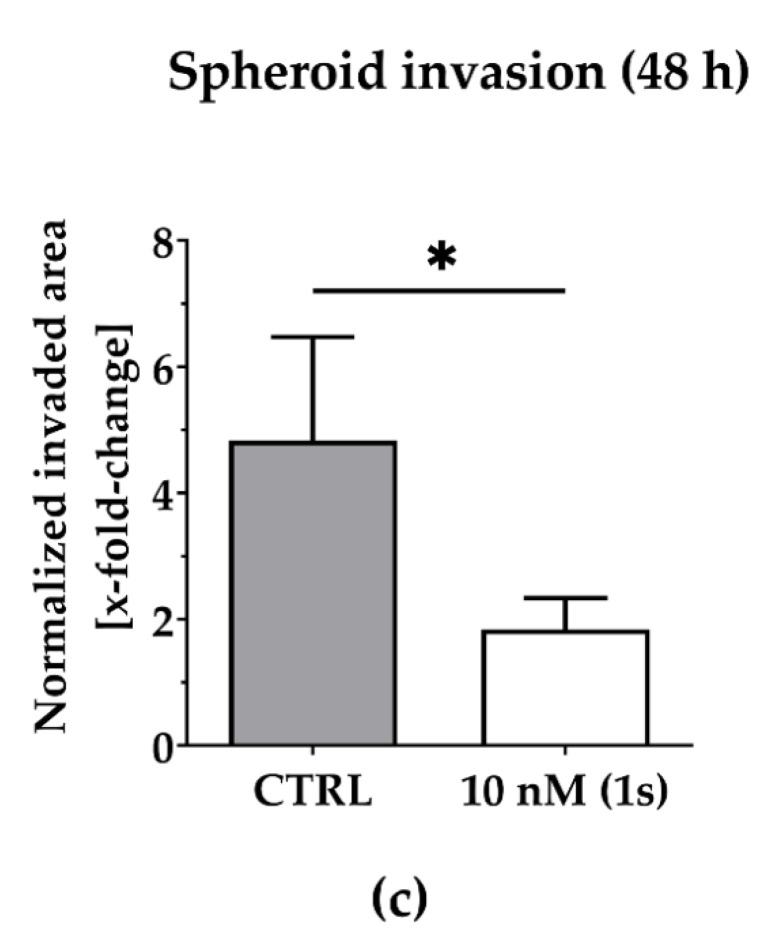
Compound (**1s**) blocks TNBC monoculture spheroid invasion in a matrigel^®^-based extracellular matrix (ECM). Monoculture spheroids were generated for 72 h before matrigel^®^ was applied for ECM formation. Spheroids were grown for another 48 h without (CTRL) or with 10 nM (**1s**). (**a**) Invaded area was measured using Fiji (open-source software [70]; version 1.52i) with manually picked positions (white squares) to determine the outline (yellow line) of the spheroid area within 0 h and after 48-h treatment. (**b**) Representative brightfield images of the monoculture spheroids. Scale bar: 500 µm. (**c**) Invaded area is calculated by normalizing the spheroid area after 48 h treatment to the spheroid size at 0 h. Statistical difference was calculated with an unpaired *t*-test (* *p* < 0.05). Columns represent the mean ± SD (*n* = 3).

To investigate the anti-migratory effects of PAs, we used TNBC monoculture spheroids composed of MDA-MB-231 cells that were grown in a matrigel^®^-based matrix. Matrigel^®^ is a commercially available mixture derived from a mouse sarcoma and comprises ECM components, such as growth factors and collagen IV, which is reported to drive motility in TNBC [69]. Over time, untreated control cells invade into the surrounding matrigel^®^ (Figure 6a). Within 48 h, the invaded area was quantified and normalized to the spheroid size at the starting time point of treatment. For untreated spheroids, the invaded area was increased 6-fold. By contrast, in the presence of compound (**1s**), invasion was significantly reduced in a dose-dependent manner (Figure 6a and Figure S4). Invasion was significantly blocked at 10 nM (**1s**) by approximately 65% (Figure 6b,c). Notably, a low-cytotoxic concentration of (**1s**) was applied to study anti-migratory effects. 

In conclusion, compound (**1s**) significantly blocks invasion of 3D TNBC monoculture spheroids.

### 2.6. Compound (***1s***) Blocks HIF-Regulated Transcription

Next, we questioned whether PAs are able to modulate hypoxia-associated HIF-activity. We evaluated HIF-mediated transcription using a second TNBC cell line, MDA-MB-468-UnaG, expressing the fluorescence protein UnaG under the control of a synthetic responsive promotor [71]. 

To simulate hypoxia, we applied CoCl_2_, which inhibits HIF-1α-hydroxylation and degradation under normoxic conditions, as described previously, thereby stabilizing HIF1-driven gene expression [72,73,74]. We confirmed hypoxia induction in MDA-MB-468-UnaG after upregulation of HIF-1α by an increase of the UnaG expression levels (Figure S6). While some studies postulate that reduced atmospheric oxygen levels do not affect cell viability [75], CoCl_2_ is an artificial system that performs a dose-dependent cytotoxicity with low-toxic effects at the concentration applied in our study (100 µM) (Figure S7a) [72]. To investigate the interplay between the NFκB and the HIF pathway under normoxic and under hypoxic conditions, we evaluated compound (**1s**) and the commercially available NFκB inhibitor BAY 11-7085, which both exhibit cytotoxicity in MDA-MB-468-UnaG (Figure S7b,d) comparable to MDA-MB-231 (Table 1 and Table S2) 

**Figure 7:**
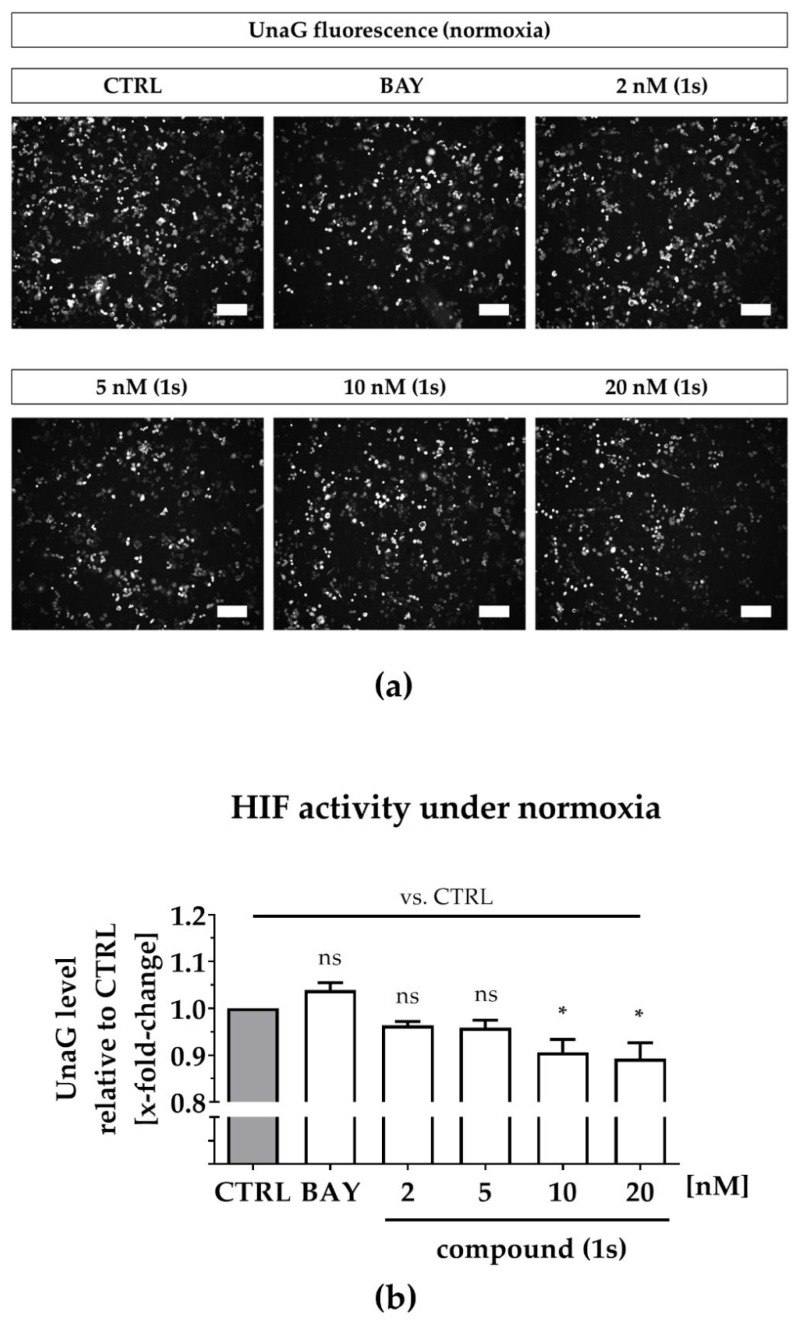

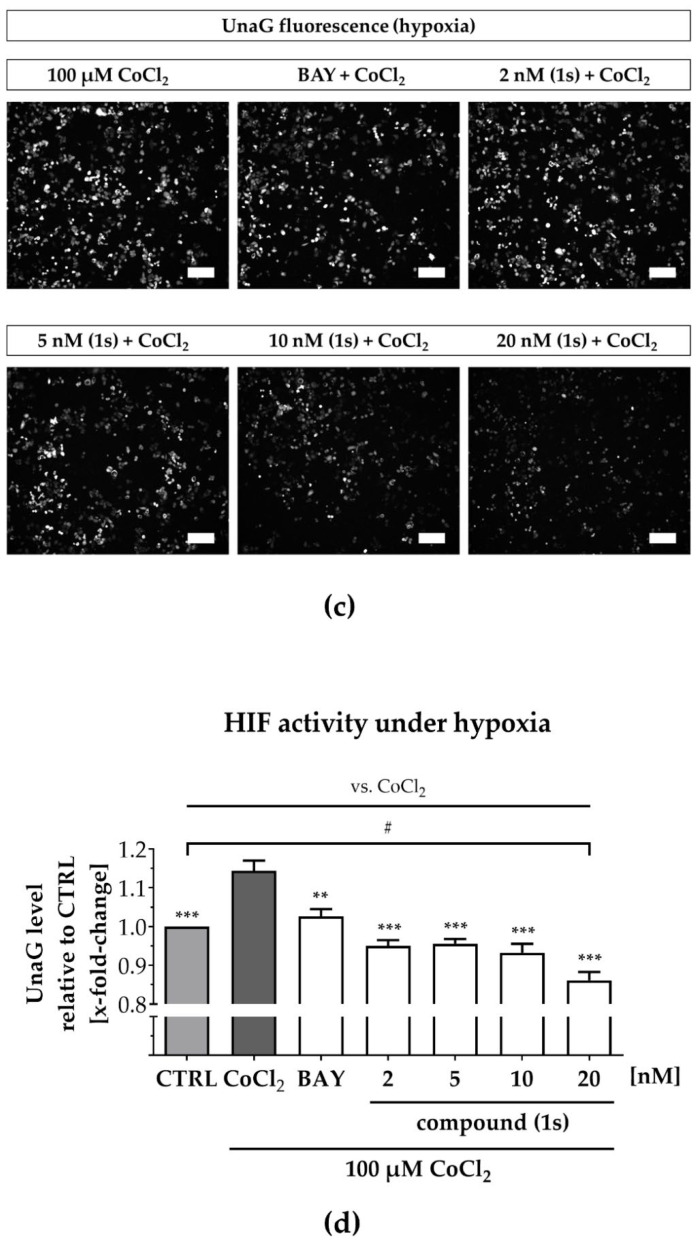
Compound (**1s**) decreases HIF-dependent gene transcription. MDA-MB-468-UnaG cells were pre-treated for 1 h without (CTRL) or with compound (**1s**) or BAY 11-7085 (BAY) before being grown (**a**,**b**) in the absence (normoxia; CTRL) or (**c**,**d**) presence of 100 µM CoCl_2_ (hypoxia) for another 24 h. (**a**,**c**) Representative fluorescence images. Scale bar: 200 µm (**b**,**d**) Total fluorescence intensity of HIF-mediated UnaG was normalized to the CTRL group. Statistical difference was calculated with one-way ANOVA (**b**) vs. CTRL (normoxia) (ns = not significant, * *p* < 0.05). (**d**) vs. CTRL (hypoxia) (# *p* < 0.05); vs. CoCl_2_ (* *p* < 0.05, ** *p* < 0.01, *** *p* < 0.001). (**b**,**d**) Columns represent the mean ± SEM (*n* = 3).

Under normoxia, compound (**1s**) significantly decreased HIF-1α-mediated UnaG expression by 11 ± 3% at 10 nM and by 13 ± 4% at 20 nM, while BAY 11-7085 had no significant effects (Figure 7a,b). When cells were pre-treated with compounds before CoCl_2_-simulated hypoxia, the increase in HIF-1α-activity was blocked in the presence of 6 µM BAY 11-7085 as well as by 2 nM, 5 nM, 10 nM and 20 nM of compound (**1s**)**.** Moreover, (**1s**) significantly reduced HIF-1α-activity by 14 ± 4% at 20 nM (Figure 7c,d).

A reduction in HIF-1α-activity was solely found for compound (**1s**), and maintained under normoxic as well as under hypoxic conditions. However, CoCl_2_-induced HIF activity was blocked by both NFκB-inhibiting compounds (**1s**) and BAY 11-7985, indicating that the NFκB-pathway might be involved in CoCl_2_-simulated hypoxia. In conclusion, PAs may present a class of compounds that have multiple targets, including players in the inflammation-associated NFκB-pathway and the hypoxia-associated HIF-pathway in TNBC.

### 2.7. Compound (***1s***) Inhibits NFκB Activation Via IκBα Stabilization

To further explore the NFκB transcriptional blockade, we evaluated the upstream regulation of NFκB via analysis of the NFκB inhibitor IκBα. Using a second TNBC cell line, MDA-MB-468-UnaG, we evaluated TNFα-mediated IκBα degradation under normoxic and hypoxic conditions. 

**Figure 8:**
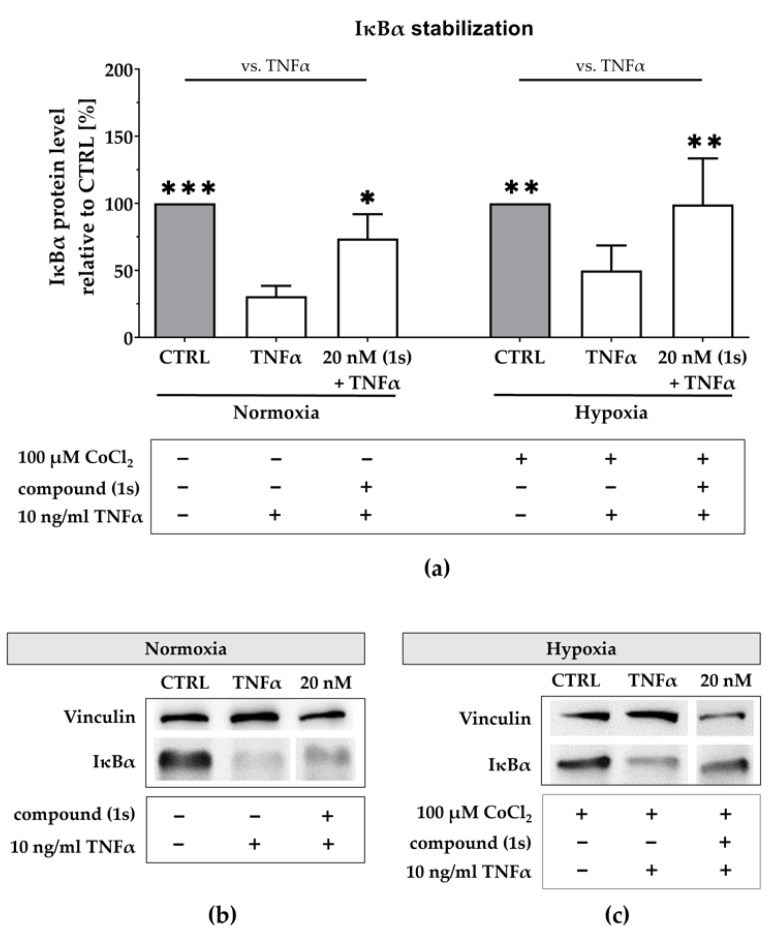
Compound (**1s**) stabilizes IκBα under normoxic and under hypoxic conditions. (**a**,**c**) For hypoxia studies, MDA-MB-468-UnaG cells were exposed to 100 µM CoCl_2_ for 24 h prior to treatment. (**a**–**c**) Cells were left untreated (CTRL), stimulated with solely 10 ng/mL TNFα for 30 min (TNFα), or pre-incubated with 20 nM of compound (**1s**) for 1 h before stimulation with TNFα for 30 min. (**a**) Normalized IκBα protein level is displayed relative to CTRL. Statistical difference vs. TNFα was calculated with two-way ANOVA (* *p* < 0.05, ** *p* < 0.01, *** *p* < 0.001). Columns represent the mean ± SD of (normoxia *n* = 3; hypoxia *n* = 2). (**b**,**c**) Representative western blot with protein lysates that are immunologically stained for IκBα and the housekeeper protein vinculin.

Under normoxia, TNFα stimulation reduced the IκBα protein level to 31 ± 6% when compared to the untreated control (Figure 8a,b). After pre-stimulation with compound (**1s**), TNFα-mediated IκBα degradation was slightly reverted. Compound (**1s**) stabilized IκBα at 20 nM when compared to the untreated control. Furthermore, we simulated hypoxia with a pre-treatment of 100 µM CoCl_2_ (Figure S6). Under simulated hypoxic conditions, TNFα stimulation significantly reduced IκBα protein level by 55 ± 22% (Figure 8a,c). When pre-stimulated with compound (**1s**), TNFα-mediated IκBα degradation is suppressed at a compound concentration of 20 nM. 

In conclusion, we confirmed NFκB blockade under hypoxic conditions for Pas, presumably by additionally suppressing HIF-activity. Based on our short time exposure we postulate that NFκB activity is not regulated on the transcriptional level. 

### 2.8. Compound (***1s***) Blocks Cell Proliferation in the G0/G1 Phase and Delays Cell Cycle Progression

In addition, we observed anti-proliferative effects for PAs in a time- and dose-dependent manner (Figures S8 and S9). Although anti-proliferative effects were observed within 24 h (Figure S9), we did not detect a shift in cell cycle population at this timepoint (data not shown). To investigate whether longer exposure could impact the cell cycle in a time-dependent manner, we extended the exposure to 48 h and 72 h. Cell populations increased in the S-phase as well as in the G2/M phase with 20 nM of (**1**) within 72 h (Figure S11).

**Figure 9:**
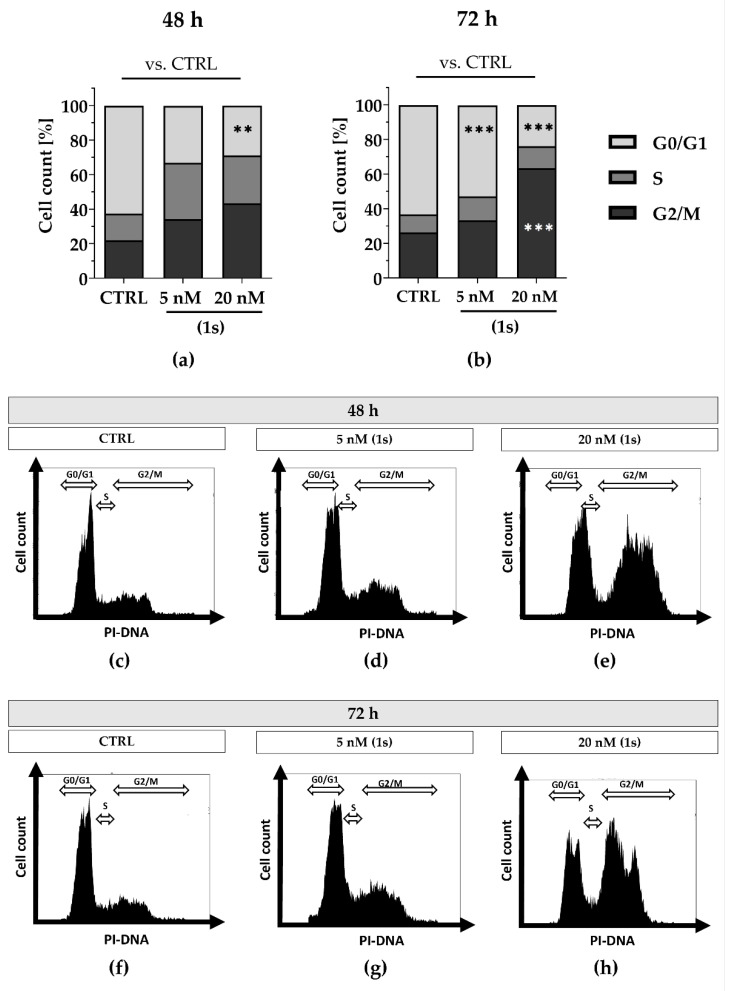
Compound (**1s**) delays cell cycle progression in a time- and dose-dependent manner. Cells were grown for (**a**,**c**–**e**) 48 h or (**b**,**f**–**h**) 72 h without (CTRL) or in the presence of (**1s**). Cell cycle phase population (G0/1; S; G2/M) was determined based on the signal of the PI-DNA complex using CytExpert. (**a**,**b**) Columns represent the mean (*n* = 3). Statistical difference was calculated using two-way ANOVA vs. CTRL (** *p* < 0.01, *** *p* < 0.001). (**b**–**h**) Representative histograms depicting the cell count within the PI-signal were illustrated with CytExpert.

To further evaluate cell cycle progression under normoxic and hypoxic conditions we utilized MDA-MB-468-UnaG cells and compared the chemically synthesized compound (**1s**) with paclitaxel. Under normoxia, we observed a time- and dose-dependent cell cycle arrest when exposed to compound (**1s**), and a dose-dependent cell cycle arrest when exposed to paclitaxel. With compound (**1s**) we confirmed a cell cycle modulation in MDA-MB-468-UnaG with a significant increase in G2/M population at 20 nM (**1s**) after 72 h (Figure 9). In agreement with already published data, paclitaxel significantly induces a G2/M arrest within 24 h ([76], Figure S12). To evaluate anti-proliferative effects under hypoxic conditions, hypoxia was mimicked in MDA-MB-468-UnaG cells with a prior exposure to 100 µM CoCl_2_. Hypoxia was previously reported to enhance G2/M arrest in MDA-MB-231 [72], and we confirmed an increase in the G2/M population in the presence of CoCl_2_ (Figure S13). Under hypoxia, neither 100 nM paclitaxel nor 20 nM of compound (**1s**) were sufficient to modulate cell cycle progression (Figure S13). We cannot exclude the possibility that the low-toxic effects of CoCl_2_ (Figure S7) may have influenced the cell cycle arrest (Figure S9). However, when comparing control cell populations to CoCl_2-_treated cells, we observed a slight increase in the G2/M population (data not shown). 

**Figure 10:**
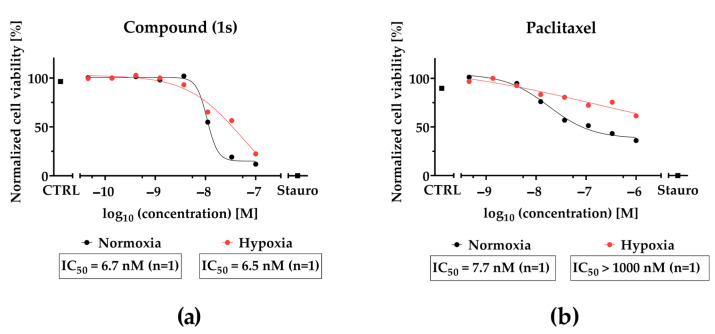
CoCl_2_-simulated hypoxia affects the inhibition of cell viability of paclitaxel but not *O*-methyltylophorinidine (**1s**) in MDA-MB-468-UnaG. For HIF-1α-stabilization, cells were grown for 24 h in the presence of 100 µM CoCl2 (hypoxia) or left unstimulated (normoxia). Compound treatment was done (**a**) for (**1s**) in a three-fold serial dilution starting at 100 nM and (**b**) for paclitaxel at a three-fold serial dilution starting at 1000 nM. Untreated (CTRL) and 10 µM staurosporine (Stauro) treated groups served as control. Cell viability was determined as relative fluorescence unit and was normalized in each individual experiment to the lowest (0%) and highest (100%) value (normalized cell viability). Data points represent one individual experiment.

Although compound (**1s**) and paclitaxel exhibit anti-tumor effects within 24 h under hypoxia (Figure 10), they do not modulate cell cycle progression within 72 h under hypoxic conditions (Figure S13). Interestingly, hypoxia strongly decreased the cytotoxic effects of paclitaxel by a factor of ≥100, whereas hypoxia barely affected the cytotoxic potential of (**1s**). Under normoxia, an increase in the sub-G0/G1 population under paclitaxel or compound (**1s**) (Figure S14) indicates DNA fragmentation, initiated through apoptosis [76]. This result suggests the induction of apoptosis through the cell cycle for compound (**1s**).

In conclusion, compound (**1s**) blocks TNBC proliferation by arresting the cell cycle at the G0/G1 state in a dose- and time-dependent manner, resulting in a delayed progression towards the S and G2 phase. Furthermore, our data indicate that in contrast to paclitaxel efficacy of *O*-methyltylophorinidine (**1s**) is not significantly reduced under hypoxic conditions.

## 3. Discussion

### 3.1. PAs Block Inflammation under Hypoxia in TNBC

Although PAs possess anti-tumor and anti-inflammatory potential in various tumor entities [77,78], their molecular mechanism in TNBC remains elusive. Thus, we explored the bioactivity of six naturally occurring derivatives, which were isolated from the plant *Tylophora ovata*: *O*-methyltylophorinidine (**1**), tylophorinidine (**2**), tylophoridicine E (**3**), 2-demethoxy-tylophorine (**4**), tylophoridicine D (**5**), and anhydrodehydrotylophorinidine (**6**). For more-in depth studies, we used the chemically prepared *O*-methyltylophorinidine (**1s**).

The tumor niche of TNBC is characterized by increased inflammation and reduced oxygen levels [24]. Hitherto, transcription blockade was reported for PAs regarding the NFκB-pathway in HepG2 [61] and the HIF-1α-pathway in T47D [62], but none of the studies included TNBC. Both, the inflammation-associated NFκB pathway and the hypoxia-induced HIF-pathway, are triggered upon paclitaxel-based chemotherapy, and are involved in mediating chemoresistance [19,24,33,79]. Chemoresistance is a major feature of cancer stem cells (CSCs) that exhibit self-renewal and activation of several pathways that prevent apoptosis and drive tumor progression via growth and invasion [30]. Thus, targeting these pathways may be a promising approach in combating TNBC, which are enriched in CSCs [31]. 

We investigated molecular targets for PAs in TNBC and found a dose-dependent inhibition of NFκB-activity that hinted toward a SAR. Blockade of NFκB via the stabilization of its inhibitor IκBα is maintained under CoCl_2_-simulated hypoxia, and blockade of HIF-regulated transcription presents PAs as multi-targeting compounds in TNBC.

Investigating TNFα- or LPS-induced NFκB transcription in TNBC, we observed a dose- and time-dependent bioactivity for compounds (**1**)–(**4**), indicating a SAR. NFκB activity was strikingly blocked within 2 h by compound (**1**) at an IC_50_ value of 17.1 ± 2.0 nM. Furthermore, although with lower potency, NFκB was blocked by (**2**) and (**4**), whereas the anti-tumor potential of (**3**) may not depend on NFκB-blockade. Although we validated the anti-tumor potential of (**3**), which was observed in nasopharyngal, lung, and colorectal cancer cells [80], and in breast cancer regarding HIF signaling [62], we hypothesize that the activity of (**3**) depends on another molecular mechanism. Compound activity clearly corresponds to the chemical structure [61,81] and for the synthetic compound (**1s**) we report similar NFκB blockade (IC_50_ = 3.3 ± 0.2 nM) and reduction of viability (IC_50_ = 4.2 ± 1 nM) compared to the natural equivalent (**1**). The initial SAR of compounds (**1**)–(**6**) reveals that bioactivity in TNBC is enhanced due to the following characteristics in their chemical structure: (I) a hydroxy moiety in the indolizidine ring at position C-14, (II) a methoxy moiety in the phenanthrene ring at positions C-3 and C-6, and (III) uncharged nitrogen in the indolizidine ring. Findings from the SAR predicted cytotoxicity in the simplified 3D TNBC co-culture model. Various studies agree with our findings [61,62,80,82] and presume that unshared electrons of the nitrogen in the indolizidine ring might be crucial for its activity [82] through interaction with proteins [63]. We validated that the positively charged nitrogen is the most critical alteration in the chemical structure resulting in activity loss, as we found in (**5**) and (**6**). Interestingly, a distinct mode of action was reported for (**5**) and (**6**) in blocking telomerase activity by promoting telomeric DNA folding into g-quadruplex at a two-digit micromolar concentration [83]. Regarding drug design, SAR is inevitable for optimization of bioactivity, and synthetic routes for compound preparation are efficient in enhancing compound yield, which is very little compared to isolation from the natural source.

Regarding NFκB-mediated transcription, studies from Gao et al. report inhibition in HepG2 cells for (**2**) and the epimer of (**1**), namely tylophorinine [61]. However, the mode of action for transcriptional blockade in TNBC remains elusive. We evaluated compound (**1s**) regarding the upstream regulation of NFκB, and found dose-dependent stabilization of the NFκB inhibitor IκBα. Because both, IκBα and NFκB are substrates of IKK, we hypothesize that PAs affect NFκB through blocking activating phosphorylation by IKK. Findings from Shiah et al. support our hypothesis regarding NFκB-inhibition in pancreatic ductal adenocarcinoma (PDAC) cancer cell line, Panc-1, using the synthetic tylophorine analogue DCB-3503. They report decreased NFκB activity through the suppression of phosphorylation by IKKα/IKKβ. As a consequence, reduced phosphorylation events of IκBα and NFκB result in the stabilization of IκBα and enhanced sequestration of NFκB in the cytoplasm to block its transcriptional activity [84]. 

NFκB activity induces drug resistance and is triggered during paclitaxel therapy [19] as well as during hypoxia [29]. The inhibition of NFκB in HepG2 [61] and inhibition of HIF-1α in T47D [62] were reported separately for PAs, e.g., (**2**). In addition, we questioned whether the anti-inflammatory potential of compound (**1s**) is maintained under hypoxic conditions. Our findings reveal a significant NFκB suppression at 20 nM via the stabilization of IκBα under CoCl_2_-simulated hypoxia. Indeed, we found modulation of HIF-mediated transcription by (**1s**) and investigated the role of NFκB inhibition on HIF-activity. Under normoxic as well as under hypoxic conditions HIF was significantly blocked at a low nanomolar concentration (20 nM). Accordingly, data from Chen et al. support our findings, reporting blockade of HIF induction under low oxygen level in the luminal breast cancer cell line T47D for tylophorinine, (**1**), (**2**), as well as for (**3**), but not for (**5**) [62]. These results match the SAR identified for our NFκB inhibition studies. NFκB blockade with the NFκB inhibitor BAY 11-7085 or compound (**1s**) was sufficient to block CoCl_2_-simulated hypoxia, whereas blockade of NFκB alone using Bay 11-7085 was not sufficient to suppress HIF, indicating that NFκB is involved in inducing CoCl_2_-induced HIF-activity, which has already been reported [85], and that PAs act in a distinct manner from the commercially available NFκB inhibitors by impacting multiple targets, e.g., NFκB and HIF in TNBC. 

The effects of PAs on the upstream signaling of both NFκB and HIF, as well as the effects on the crosstalk between both pathways, have not been examined yet. NFκB is involved in the transcriptional regulation of HIF-1α [28], and HIF-1α triggers NFκB activity by inducing nuclear translocation [29] through enhancing IKK/NFκB signaling [24]. However, it remains unclear, if (I) NFκB blockade by PAs affects HIF expression, (II) NFκB is involved in the modulation of HIF-inhibition by PAs, (III) HIF-blockade would affect NFκB inhibition by PAs. However, both pathways share triggers for activation, TNFα and CoCl_2_ [29], as well as target genes, including cyclin D1 [85]. AKT is a common upstream regulator of NFκB and HIF [86], and was reported as a target for PAs by blocking kinase activity [87]. It is poorly understood how PAs regulate phosphorylation events [84], and whether PAs act as allosteric kinase inhibitors [59,60,88]. Nevertheless, in silico docking studies postulate kinase inhibition through binding to the ATP-binding site as a pharmacological mode of action for PAs. Studies by Liu et al. report the blocking of AKT for another epimer of compound (**1**), namely HTBP1 [87], and Mostafa et al. showed blocking of Aurora A and B kinases for tylophorinine and for (**2**) [89]. However, to date, the function of kinase inhibition by PAs is unclear for breast cancer. 

In conclusion, PAs are multi-targeting compounds in TNBC with the most efficacious compounds, (**1**) and (**1s**), exhibiting a significant inhibition of IκB degradation under hypoxia. Our SAR studies for compounds (**1**)–(**6**) regarding NFκB inhibition might predict anti-tumor potential for a variety of different types of cancer. Like many other natural product families, PAs represent a compound class targeting key cell signaling pathways in drug resistance e.g., NFκB and HIF, which is distinct from their previously reported cytotoxicity. Finally, we suggest further studies to examine the distinct pharmacological target(s) in the molecular interplay of both NFκB and HIF in TNBC.

### 3.2. PAs Block Proliferation in TNBC through Arrest at the G0/G1-State and Retardation in Cell Cycle Progression

Proliferation is a multi-step process comprising the interphase (G1, S, G2) and mitosis (M) while non-proliferative cells are resting at a G0 state. Cell cycle phase transition is regulated by the balance of cyclins as well as cyclin dependent kinases and checkpoint proteins controlling DNA damage and chromosomal segregation, as well as modulating cell fate toward progression or apoptosis [90]. Although the anti-proliferative effects of PAs were reported for breast cancer cell lines, including MDA-MB-231 [65], the regulation of the cell cycle remains unclear in TNBC. In our studies, the anti-proliferative behavior of compound (**1**) was observed in TNBC (MDA-MB-231). For PAs, effecting the cell cycle arrest at the G1 or S-phase is reported for various cancer entities [60,68,91,92]. Cell cycle arrest results from the downregulation of cell cycle-related cyclins [93] and the subsequent targets were reported for PAs in the following ways: decrease of cyclin D1 (G1-progression) by e.g., (**2**) [61], decrease of cyclin E1 (G1/S-transition) by the tylophorine analogue DCB-3503 [88], or decrease of cyclin A2 (G1/S; S/G2-transition) by tylophorine [92]. To investigate modulation of the cell cycle state in TNBC, we tested the synthetically prepared compound (**1s**) and found a time- and dose-dependent arrest at the S- and G2-phase, presumably through delay in G1/S transition. The delay in G1-progression [93] might rely on the downregulation of cyclin D1 by targeting NFκB [61]. NFκB blocking was also reported for the tylophorine analogue in pancreatic [84] and hepatocellular [61] cancer cell lines, and thus we hypothesize a common mode of action regarding proliferation blocking in diverse cancer entities. In agreement with our findings, there was no shift detected in the cell cycle population within 24 h when exposing T47D cells to tylophorine [68]. This indicates that within 24 h cells might arrest at the G1-state and delay cell cycle progression. In contrast, studies by Gao et al. claimed a cell cycle arrest to be cancer type dependent [60].

Overall, the mechanism of PAs in cell cycle modulation differs significantly from the M-phase inhibitor paclitaxel, known to arrest progression at the G2/M state by preventing depolymerization of mitotic spindles during cell division [94]. In contrast, proliferation blocking by PAs might rely on inhibiting checkpoint proteins in the cell cycle or by inhibiting DNA replication. Recently, Aurora A and B kinases were found as novel targets for tylophorinine and for (**2**) at a low micromolar concentration in MCF7 [89]. As a direct substrate, p53 is phosphorylated and subsequently degraded [95]. Downregulation of p53 and its target genes coding for p21 was also reported for tylophorine and its analogue DCB-3503 [60,88] while studies by Gao et al. assume that cell cycle arrest is independent of p53 activity and thus DNA damage [60]. Furthermore, the blocking of DNA replication was reported for (+)-(13aS)-deoxytylophorinine (the stereoisomer of (**4**)), through intercalation into nucleic acid [63,64], and for tylophorinine and (**2**) through the blocking of the nucleic acid synthesis [96,97]. Preferably, (+)-(13aS)-deoxytylophorinine intercalates at AT-rich sequences, which are mainly found upstream of the transcription start site [63,64]. Functional groups at the phenanthrene ring are suggested to be important for binding to nucleic acid [63] and might explain general blocking of protein synthesis [98]. On the contrary, findings by Wang et al. assume that reduced DNA replication is a side effect of downregulating proteins involved in DNA synthesis [99].

In conclusion, cell cycle modulation in TNBC by PAs is distinct from the standard-of-care agent paclitaxel, presumably by blocking DNA replication and blockade or downregulation of proteins involved in cell cycle transition, with all events resulting in G0/G1-arrest to delay cell cycle progression. The mechanism of cell cycle modulation may be different in different solid cancer entities. 

### 3.3. PAs Maintain Anti-Tumor Potential in a 3D Co-Culture Model Comprising Murine CAFs and Block TNBC Invasion in a 3D Monoculture Spheroid

As drug efficacy differs in monolayer cells compared to the respective 3D biology [100,101], we characterized compound (**1**)/(**1s**) in a 3D TNBC spheroid model. Spheroids represent a predictive preclinical high-throughput model for solid tumors by reflecting the physiological conditions within the TME that drive drug resistance [100]. 

During spheroid formation, NFκB activity is enhanced [102] and promotes chemotherapy resistance, tumor growth, and metastasis [103]. We showed that our anti-inflammatory compounds could block tumor progression, e.g., 3D spheroid growth and invasion. Potentially, p65 knockdown studies could address the involvement of NFκB in TNBC progression in more molecular detail. Migration as an early step of metastasis is induced by NFκB-mediated downregulation of E-cadherin or upregulation of ECM-proteolytic enzymes, such as matrix metalloprotease (MMP)-9 and EMT-associated markers, e.g., vimentin and snail1 [104]. In MDA-MB-231 cells, NFκB was reported to enhance migratory behavior [19] and findings from Sperlich and Teusch report suppression of TNBC invasion by blocking NFκB signaling [23]. Based on these findings, we questioned whether anti-inflammatory (**1s**) is sufficient to suppress block TNBC invasion. So far, anti-migrative behavior in cancer cells has been reported for tylophorine [105] and for the tylophorine analogue, NK007 [91]. To our knowledge, migration or invasion studies of PAs have hitherto been conducted in vitro using 2D cell monolayers not reflecting the ECM composition and stiffness. In our study, we investigated compound (**1s**) regarding the invasion of TNBC monoculture spheroids into a matrigel^®^-based matrix and found a significant invasion blockade at 10 nM, presumably by blocking NFκB. Indeed, NFκB inhibition by PAs correlates with reduction of MMP-9 and reduced migration [105]. Interestingly, novel targets of PAs, Aurora A and B kinases, are also involved in mediating EMT via activation of NFκB [95,106]. However, to date, no in vitro studies have included the role of PAs in the crosstalk of Aurora A/B and NFκB. However, as drug resistance is a major cause of therapy failure, targeting NFκB-regulated EMT, which is a key process for maintaining cancer stemness and drug resistance [30], has promising potential in TNBC therapy for recurrent cancer. 

Our 3D TNBC monoculture model may not fully reflect physiological conditions, thus we aimed to investigate the anti-tumor potential of (**1**) by mimicking the TNBC TME by inclusion of tumor-specific stromal cells and ECM-matrix components [107]. Spheroids were grown with additional type I collagen, the main component in the stroma of TNBC patients [108], mainly secreted by CAFs [43] and elevated in breast cancer [109]. Regarding cytotoxicity, the anti-tumor potential of PAs in the 3D co-culture spheroids was predictable based on the SAR studies for NFκB inhibition with (**1**) and (**1s**) showing striking reduction of cell viability at an IC_50_ of 21.7 ± 2.5 nM and 11.2 ± 2.1 nM, respectively.

The hypoxic and immunosuppressive TME is linked to enhanced inflammation [25,29,39,46], and NFκB as well as HIF are crucial to maintaining tumor-promoting features of CAFs. CAFs are not targeted by paclitaxel, whereas bioactive PAs (**1**)–(**4**) maintain dose-dependent anti-proliferative effects presumably by blocking NFκB and HIF signaling and reducing tumor-promoting CAF activation. Because αSMA positive CAFs have been widely described as tumor-promoting and correlating with worse prognosis [44,110], we assume that mainly paracrine signaling enhances inflammation [37], i.e., NFκB, which might lead to increased vulnerability of co-culture spheroids to treatment with NFκB-inhibiting PAs. 

### 3.4. Compound (***1***)/(***1s***) Exhibits Superior Anti-Tumor Potential Compared to the Chemotherapeutic Agent Paclitaxel

Compound (**1**)/(**1s**) exhibits superior anti-tumor potential in TNBC compared to paclitaxel regarding cytotoxicity in 2D monolayer cells as well as in 3D co-culture spheroids. Furthermore, (**1**)/(**1s**) is more efficacious under hypoxia. 

Compound (**1**)/(**1s**) displays superior activity compared to paclitaxel by targeting NFκB and HIF, which are both involved in paclitaxel resistance [20]. Under hypoxia, the bioactivity of compound (**1**) was barely affected and maintains NFκB blockade via the stabilization of the IκBα inhibitor, presumably by additionally targeting HIF. By contrast, paclitaxel loses cytotoxic potential under hypoxia, presumably due to HIF-related resistance [111]. For paclitaxel, a reduced induction of apoptosis was reported by diminishing the arrest in the G2/M cell cycle state under hypoxia [79]. Under hypoxia as well as under normoxia paclitaxel induces autophagy [112], potentially mediating paclitaxel resistance [113]. On the other hand, the blockade of the HIF-pathway, a driving force for drug resistance, was reported to sensitize tumor cells to paclitaxel [33]. Hypoxia is also involved in enhancing NFκB activity and blocking both pathways, which might be sufficient to suppress tumor progression [24].

Although our data indicate that the pharmacological mode of action for NFκB blockade by PAs might be different from the underlying mode of action resulting in proliferation inhibition, further research needs to be done to develop novel analogues with reduced cytotoxicity while maintaining the NFκB blocking potential. A major obstacle for PAs in clinical application is the neurotoxicity of these compounds. To date, in vivo activity in breast cancer has been described for cryptopleurine, but a clinical trial in phase I was discontinued due to central nervous system (CNS) toxicity [114]. In contrast, the general cytotoxicity of PAs was excluded on an epithelial breast cell line MCF10A in vitro for the compound 3-*O-*demethyltylophorinidine [115]. In line with this, Ueno et al. hypothesize that a lack of hydroxy moiety at the indolizidine ring increases anti-tumor behavior while reducing its cytotoxicity in vivo [98]. High neurotoxicity and contradictory findings emphasize the relevance of extended SAR studies to optimize drug specificity. 

In summary, the compound (**1**) exhibits superior anti-tumor potential compared to paclitaxel and maintains efficacy under hypoxia. Further studies might include combinational treatments of PAs specifically addressing the resistance development of paclitaxel. Nevertheless, SAR studies and drug design are inevitable in optimizing drug efficacy for clinical application.

## 4. Materials and Methods

### 4.1. Cell Culture and Compounds

All cell lines were grown and incubated in a humidified 5% CO_2_ atmosphere at a constant 37 °C. The TNBC cell line MDA-MB-231 was obtained from the European Collection of Authenticated Cell Cultures (ECACC, Salisbury, UK). Subculture was performed in RPMI 1640 medium (Thermo Fisher Scientific, Schwerte, Germany; catalogue number (#)21875-034) supplemented with 15% FCS (Thermo Fisher Scientific; #10270-106) and 1% PS (Thermo Fisher Scientific; #15140-122). The MDA-MB-231 cell line containing a plasmid with the NFκB response element and the gene sequence for the nanoluc-luciferase protein (NFκB-MDA-MB-231-nanoluc) was generated as described [47]. Subculture and cell clone selection was performed in high glucose DMEM (Thermo Fisher Scientific; #41966-029) supplemented with 10% FCS (Thermo Fisher Scientific; #10270-106), 1% PS (Thermo Fisher Scientific; #15140-122), and 400 µg/mL hygromycin B (Thermo Fisher Scientific; #10687010). Another MDA-MB-231 cell line expressing the NFκB-reporter gene firefly-luciferase (NFκB- MDA-MB-231-firefly) was purchased from Signosis (Santa Clara, CA, USA; #SL-0043). Subculture and cell clone selection was performed in high glucose DMEM (Thermo Fisher Scientific; #41966-029) supplemented with 10% FCS (Thermo Fisher Scientific; #10270-106), 1% PS (Thermo Fisher Scientific; #15140-122), and 100 µg/mL hygromycin B (Thermo Fisher Scientific; #10687010). Starvation medium used for the NFκB inhibition assay was reduced to 1% FCS. The bulk-selected TNBC line MDA-MB-468-UnaG was generated according to the published protocol [71]. In brief, cells were stably transfected with a plasmid containing a HIF-1 responsive element regulating the expression of the fluorescence protein UnaG. Subculture and cell clone selection was performed in RPMI 1640 supplemented with 10% FCS (Thermo Fisher Scientific; #10270-106), 1% PS (Thermo Fisher Scientific; #15140-122), and 1 mg/mL G418 disulfate (Roth, Karlsruhe, Germany; #CP11.2). The primary murine CAFs were isolated as described [116]. In brief, CAFs were isolated from 4T1 mammary tumor-bearing BALB/c Ub-GFP mice (BALB/c mice expressing GFP under the human ubiquitin C promoter). Subculture of CAFs was performed in high glucose DMEM supplemented with 10% FCS (Thermo Fisher Scientific; #10270-106), and 1% PS (Thermo Fisher Scientific; #15140-122).

The alkaloids (**1**)–(**6**) and (**1s**), paclitaxel (Santa Cruz Biotechnology, Heidelberg, Germany; #sc-201439), BAY 11-7085 (Hycultec, Beutelsbach, Germany; #HY-10257-10), and staurosporine (Sigma-Aldrich, Taufkirchen, Germany; #S5921) were dissolved in 100% dimethyl sulfoxide (DMSO; Roth; #A994.1) to a final concentration of 10 mM. Subsequent dilutions were performed in cell culture medium with a maximal concentration of 0.1% DMSO. For dose–response experiments, the compounds were serially diluted in medium at a 10× concentration on a V-bottom 96 well plate (Greiner, Frickenhausen, Germany; #651180). Cobalt (II) chloride (CoCl_2_; Sigma-Aldrich; #C8661) was dissolved in PBS (Thermo Fisher Scientific; #14190). Cell seeding and compound injection was partly performed using the CyBio^®^ robot (Analytik Jena, Jena, Germany). 

### 4.2. Extraction and Purification of a Phenanthroindolizidines Alkaloids Library

Dried plant parts of *Tylophora ovata* (*T. ovata*), including roots and aerial parts, were bought from Yulin Traditional Chinese Medicine Market. The plant had been collected in Guangxi Province, China in 2017 and was identified by Ms. Zhou (Junhao Traditional Chinese Medicine Company). The air-dried powdered plant material of T. ovata (3.2 kg) was extracted with MeOH (pH was adjusted to 10–11 by adding 25% NH_3_∙H_2_O), followed by concentration in vacuo to afford the crude extract. The latter was then subjected to liquid–liquid partitioning between H_2_O (pH was adjusted to 2 by addition of 0.5 M HCl) and EtOAc, yielding the EtOAc phase and the acidic aqueous phase. The obtained aqueous phase was basified by addition of 25% NH_3_∙H_2_O to pH 10, and extracted with ethyl acetate, yielding the alkaloid-containing EtOAc extract (1.11 g). The EtOAc extract was then loaded in a C18 reversed phase column and eluted successively with H_2_O -MeOH to yield 9 fractions. Based on HPLC analysis, fraction 8 (200 g), which eluted with 90% and 100% MeOH, was subjected to further separation over Sephadex LH-20 and eluted with MeOH to give 6 subfractions. Purification of subfraction 8.3 (62 mg) by semipreparative HPLC with MeOH and 0.1% HCOOH in H_2_O as eluent (from 35% to 100% MeOH, 15 min) yielded *O*-methyltylophorinidine ((**1**), 7.1 mg), tylophorinidine ((**2**), 8.3 mg), tylophoridicine E ((**3**), 1.4 mg), and 2-demethoxytylophorine ((**4**), 2.2 mg). In the same manner, purification by semipreparative HPLC of subfraction 8.4 (20 mg) yielded tylophoridicine D ((**5**) 1.5 mg) and anhydrodehydrotylophorinidine ((**6**), 1.0 mg).

*O*-Methyltylophorinidine (**1**): 379 g/mol; brown, solid, *m/z* 380 [M + H]^+^, UV, and ^1^H NMR data were identical to those reported in the literature [117].

Tylophorinidine (**2**): 365 g/mol; brown, solid, *m/z* 366 [M + H]^+^, UV, and ^1^H NMR data were identical to those reported in the literature [117].

Tylophoridicine E (**3**): 365 g/mol; brown, solid, *m/z* 366 [M + H]^+^, UV, and ^1^H NMR data were identical to those reported in the literature [82].

2-Demethoxytylophorine (**4**): 363 g/mol; brown, solid, *m/z* 364 [M + H]^+^, UV, and ^1^H NMR data were identical to those reported in the literature [117].

Tylophoridicine D (**5**): 360 g/mol; brown, solid, *m/z* 360 [M ]^+^, UV, and ^1^H NMR data were identical to those reported in the literature [82].

Anhydrodehydrotylophorinidine (**6**): 346 g/mol; brown, solid, *m/z* 346 [M ]^+^, UV, and ^1^H NMR data were identical to those reported in the literature [83,118].

### 4.3. Chemical Synthesis of O-Methyltylophorinidine (***1s***)

**Scheme 1:**
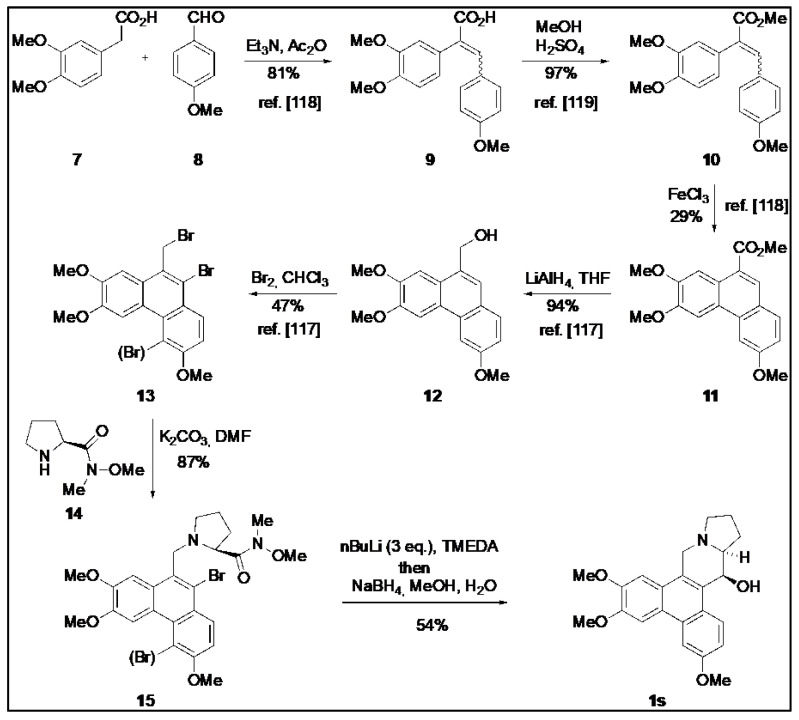
Synthesis of *O*-methyltylophorinidine (**1s**). The procedure for compound (**1s**) synthesis was adapted from Wang et al. (2010) [119], Wang et al. (2008) [120] and Su et al. (2008) [121].

O-Methyltylophorinidine (**1s**) was synthesized according to the strategy of Wang et al. [119] as summarized in Scheme 1. The bromide 13 (mixture) was prepared following published protocols [120,121] and then converted to (**1s**) as detailed below.

The mixture of bromides **13** [119] (0.143 g, ca. 0.3 mmol, 1.0 eq.) was dissolved in 20 mL of DMF and the (S)-proline-derived Weinreb amide **14** (0.092 g, 0.34 mmol, 1.13 eq.) and K_2_CO_3_ (0.193 g, 1.4 mmol, 4.7 eq.) were added. The mixture was heated at 160 °C for 7.5 h (reflux) before the deep green-black solution was allowed to cool to room temperature overnight. After the addition of H_2_O, the aqueous phase was extracted with EtOAc until the organic phase remained colorless. The combined organic phases were dried over MgSO_4_ and concentrated in vacuo. Purification by column chromatography (SiO_2_, cyHex/EtOAc 1:5) yielded a mixture of mono- and dibrominated substitution products **15** (0.145 g ca. 0.26 mmol, 87%) as a voluminous gray foam which was employed as such in the next step.

**Figure 11:**
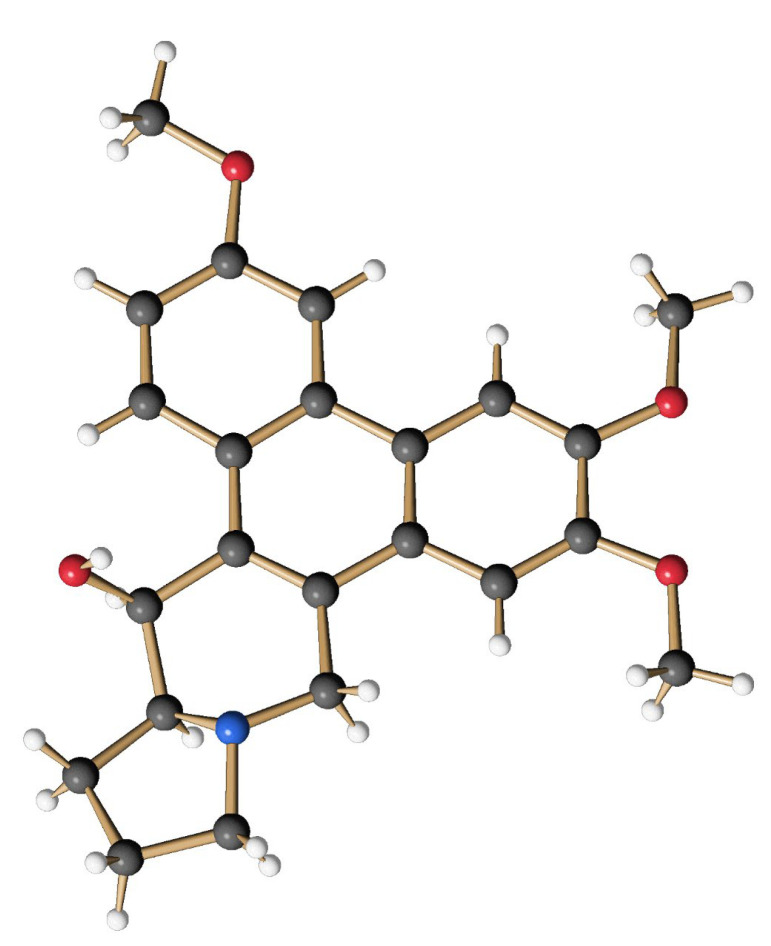
Structure of the synthetic *O*-methyltylophorinidine (**1s**) in the crystalline state.

The obtained mixture of bromides (**15**) (0.285 g, 0.499 mmol) was dissolved in 30 mL of anhydrous THF, and the yellow solution was cooled to −78 °C before 0.28 mL of TMEDA (1.89 mmol, 3.7 eq.) and 0.73 mL of a solution of *n*BuLi (2.2 N in hexane, 1.62 mmol, 3.2 eq.) were successively added under an atmosphere of argon. The intensely orange-colored solution was stirred at −78 °C for 4 h and then allowed to warm to −45 °C. At this temperature, 11 mL of MeOH and 0.19 g (5 mmol, 10.0 eq.) of NaBH_4_ were added. After 1 h the cold bath was removed and stirring was continued for 19 h at room temperature to give an intense yellow suspension. After the addition of 10 mL of H_2_O, the aqueous phase was extracted several times with CH_2_Cl_2_. The combined organic phases were washed with H_2_O and brine, and dried over MgSO_4_ before the solvent was removed in vacuo. The crude product (light yellowish fine crystals) was purified by column chromatography on silica using CH_2_Cl_2_/MeOH/Et_3_N (50:1:0.1) as an eluent. The product-containing fractions were concentrated and purified again by column chromatography using cyHex/EtOAc (1:5) as eluent. After the removal of the solvent, 102 mg (0.27 mmol, 54 %) of O-methyltylophoriniodine (**1s**) was obtained, a light yellow, fine crystalline solid. **m_p_:** 231–233 °C; **^1^H NMR** (CDCl_3_, 500 MHz, 296 K): *δ* 1.90 (m, 2H, H-12 und H-13), 2.02 (m, 1H, H-12), 2.21 (m, 1H, H-11), 2.35 (m, 1H, H-13a), 2.42 (m, 1H, H-13), 3.03 (d, *J* = 14.8 Hz, 1H, H-9), 3.28 (t, *J* = 8.1 Hz, 1H, H-11), 3.40 (d, *J* = 14.8 Hz, 1H, H-9), 3.82 (s, 3H, OMe-7), 4.05 (s, 3H, OMe-3), 4.10 (s, 3H, OMe-6), 4.93 (d, *J* = 2.4 Hz, 1H, H-14), 5.30 (br, 1H, OH-14), 6.19 (s, 1H, H-8), 7.27 (dd, *J* = 9.1 Hz, 2.5 Hz, 1H, H-2), 7.58 (s, 1H, H-5), 7.75 (d, *J* = 2.5 Hz, 1H, H-4), 8.44 (d, *J* = 9.1 Hz, 1H, H-1). ${\left[\alpha \right]}_{D}^{20}$= + 88.9° (c = 0.066, CHCl_3_); **HRMS** (ESI): calc. for [M+H]^+^: 380.1856; found: 380.1855; calc. for [M+Na]^+^: 402.1676; found: 402.1675. The analytical data are in agreement with those given in the literature [62]. The structure of (**1s**) was additionally secured by X-ray crystal structure analysis (Figure 11).

### 4.4. Nuclear Factor Kappa B (NFκB) Inhibition Studies

#### 4.4.1. NFκB Inhibition Assay (2 h)

Experiments were performed according to the published protocol [47]. On day 1, 4 × 10^4^ NFκB-MDA-MB-231-nanoluc cells were seeded into white 96 well plates (Greiner; #655098). On day 2, the medium was replaced with starvation medium and the cells were pre-incubated for 20 min with either 100 µL of DMSO (Roth; #A994.1) alone at a concentration of maximal 0.1% or with the final concentration of the diluted compounds (Table 3). To activate NFκB signaling, cells were stimulated with 1 µg/mL LPS (Sigma-Aldrich; #L2630) beforehand. NFκB-dependent luciferase activity was measured according to the manufacturer`s protocol for the Nano-Glo Luciferase Assay System (Promega, Mannheim, Germany; #N1110). NFκB-dependent luminescence was determined as relative light units (RLU) using the microplate reader Spark^®^ (Tecan, Männedorf, Switzerland).

#### 4.4.2. NFκB Inhibition Assay (24 h)

On day 1, 3 × 10^4^ NFκB MDA-MB-231-firefly cells were seeded into white 96 well plates (Greiner; #655098). On day 2, the medium was replaced with starvation medium and the cells were pre-incubated for 20 min with either 100 µL of DMSO (Roth; #A994.1) alone at maximal 0.1%, or with the final concentration of the diluted compounds (Table 3). To activate NFκB signaling, cells were stimulated with 20 ng/mL TNFα (Peprotech, Hamburg, Germany; #300-01A). Cell lysis was performed in 20 µL 1× cell lysis buffer according to the manufacturer’s instruction for the Luciferase Assay System (Promega #E1500). NFκB-dependent luminescence was determined as relative light units (RLU) using the microplate reader Spark^®^ (Tecan).

### 4.5. 2D Cell Viability

Cell viability was determined based on resazurin-reduction in viable cells according to the PrestoBlue^TM^ Assay. On day 1, MDA-MB-231 (1.5 × 10^4^ cells per well or 7.5 × 10^3^ cells per well) were seeded into black 96 well plates (Greiner; #655090) in a total volume of 100 µL. On day 2, the compounds or DMSO (Roth; #A994.1) alone at maximal 0.1% were applied (final concentrations after serial dilution shown in Table 3). An amount of 20 µM staurosporine (Sigma-Aldrich; #S5921) served as a positive control. On day 3, the PrestoBlue^TM^ reagent (Invitrogen^TM^, Darmstadt, Germany; #A13262) was applied according to the manufacturer`s instructions. Finally, cell viability-dependent fluorescence was recorded in relative fluorescence units (RFU) with excitation/emission (Exλ/Emλ) at 535/590 nm or 560/590 nm, using the microplate reader Spark^®^ (Tecan).

### 4.6. 3D Co-Culture Studies

To emulate the TNBC microenvironment, 3D spheroids composed of MDA-MB-231 and CAFs at a ratio of 1:2 in a collagen-based matrix were generated. The culture medium of both cell lines was mixed (DMEM:RPMI 1640 (1:1) + 12.5% FCS + 1% PS) and supplemented with 0.05 mg/mL collagen type I (Thermo Fisher Scientific; #A1048301). On day 1, 3000 cells were seeded into a low-attachment 96 well plate (Greiner; #650970). Spheroids were grown for 72 h before proceeding with compound treatment on day 4, and analysis on day 7 according to the “Spheroid immunostaining”, “3D cell viability”, or “3D growth” studies presented in the results section.

#### 4.6.1. Spheroid Immunostaining

To analyze the cellular distribution within the spheroid, MDA-MB-231 were identified based on the proliferation marker Ki67 [122], and the primary murine CAFs were stained with the specific marker for activated myofibroblast α-smooth muscle actin (αSMA) [123] according to our previous work [124]. Co-culture spheroids were generated for 72 h before being grown for another 72 h either without or in the presence of 100 nM paclitaxel (Santa Cruz Biotechnology; #sc-201439) or 100 nM compound (**1**). Spheroids were fixed with 4% (*w*/*v*) paraformaldehyde (Sigma-Aldrich; #0335.2) dissolved in 1× PBS (Thermo Fisher Scientific; #14190), pH 7.4) and rinsed three times in 1 mL of 3 mg/mL polyvinylpyrrolidone (PVP; Sigma-Aldrich; #P0930) dissolved in 1× PBS (PBS/PVP). Prior to antibody staining, spheroids were permeabilized with 0.25% (*v*/*v*) Triton™ X-100 (Sigma-Aldrich; #X100), dissolved in PVP/PBS, and blocked for 1 h in blocking solution (0.1% (*w*/*v*) bovine serum albumin (BSA; Roth; #8076.2), 0.01% (*v*/*v*) Tween 20 (Roth; #9127.1) dissolved in 1× PBS. 

Primary antibodies against α-smooth muscle actin (αSMA) (Sigma-Aldrich; #A5228; mouse IgG) and against Ki67 (R&D Systems, Wiesbaden, Germany; #MAB7617; rabbit IgG) were diluted in blocking solution and incubated overnight at 4 °C. The secondary antibody solution contained 1:500 (*v*/*v*) DAPI (Sigma-Aldrich; #D9542; 1 mg/mL in double distilled water) and the species-specific antibodies in a dilution of 1:200 (*v/v*): donkey-anti-mouse (Invitrogen^TM^; #A31571; AF 647 conjugate) and donkey-anti-rabbit (Invitrogen^TM^; #A10040; AF 546 conjugate). Finally, spheroids were optically cleared with dehydration in increasing ethanol concentrations, followed by a 1 h incubation in benzyl alcohol (Alfa Aesar, Kandel, Germany; #L03258) and benzyl benzoate (Alfa Aesar; #L03292) in a ratio of 1:2 (*v*/*v*) (BABB). Cleared spheroids were then transferred to a black flat-bottom 96 well plate (Ibidi, Gräfelfing, Germany; #89626). Samples could be stored at 4 °C until analysis with the laser scanning microscope (LSM)880 AxioObserver (Zeiss). Z-stacked images of the spheroids were recorded at 200× *g* magnification with excitation/emission wavelength at 405/442 nm, 561/588 nm, and 633/703 nm.

#### 4.6.2. 3D Cell Viability Studies

Cell viability was determined based on the ATP content in viable cells according to the manufacturer`s instructions (Promega #G9682). Co-culture spheroids were generated for 72 h before they were grown for another 72 h with or without exposure to the compounds. Twenty µM staurosporine (Sigma-Aldrich; #S5921) served as a positive control. Cell viability-dependent luminescence was determined as relative light units (RLU) using the microplate reader Spark^®^ (Tecan).

#### 4.6.3. 3D Growth Studies

Co-culture spheroids were generated for 72 h before they were exposed to either DMSO (Roth; #A994.1) alone at maximal 0.1%, 100 nM paclitaxel (Santa Cruz Biotechnology; #sc-201439), or 100 nM of compound (**1**) for another 72 h. At least three spheroids per treatment group were analyzed. For that purpose, brightfield images of the spheroids were acquired at 100× *g* magnification using the microscope Axio Vert.A1 (Zeiss). The spheroid surface area was quantified in brightfield images using Fiji (open-source software [70]; version 1.52i) and was further defined as spheroid size. In each individual experiment spheroid size was normalized to the smallest (which was set to 0%) and to the largest spheroids (which was set to 100%).

### 4.7. 3D Invasion Studies

To study TNBC invasion in an extracellular matrix (ECM)-like environment, mono-culture spheroids of MDA-MB-231 were generated in matrigel^®^, as described [23]. In brief, 3000 MDA-MB-231 cells were seeded in 0.25% (*v*/*v*) matrigel^®^ (Corning, Taufkirchen, Germany; #356432) into a low-attachment 96 well plate (Greiner; #650970 or Cenibra, Bramsche, Germany; #ULA-96U). For cell aggregation, cells were centrifugated for 10 min at 136× *g* at 4 °C before being grown for 72 h. Invasion was allowed for another 72 h before analyzing at least three spheroids per treatment group. For that purpose, brightfield images of the spheroids were acquired at 50× *g* magnification using the microscope Axio Vert.A1 (Zeiss). The spheroid area before treatment and the spheroid area including the invaded area after compound treatment were quantified in brightfield images using Fiji (open-source software [70]; version 1.52i). 

### 4.8. Hypoxia Studies

#### 4.8.1. HIF Inhibition

The HIF activity after treatment with compound (**1s**) or with the commercially available NFκB inhibitor BAY 11-7085 ((Hycultec, Beutelsbach, Germany; #HY-10257-10),) for 24 h was determined using the UnaG-reporter gene cell line MDA-MB-468-UnaG. On day 1, 1.5 × 10^4^ MDA-MB-468-UnaG cells were seeded into a black 96 well plate (Greiner; #655090). On day 2, the cells were pre-treated for 1 h with the compounds or DMSO (Roth; #A994.1) alone at maximal 0.1% before starting a growing period for another 24 h in the absence (normoxia) or in the presence of 100 µM CoCl_2_ (hypoxia) (Sigma-Aldrich; #C8661). HIF-mediated UnaG expression was recorded in z-stacked images using the CQ1 high-content imaging system (Yokogawa, Wehr, Germany; 100× *g* magnification). Beforehand, the UnaG fluorescence intensity was determined in UnaG-expressing cell clones (Figure S5).

#### 4.8.2. Western Blot

We investigated NFκB-blocking by determining the protein expression of its cytoplasmic inhibitor IκBα in MDA-MB-468-UnaG cells. On day 1, the cells were seeded into a 6 well plate (Greiner; #657160). On day 2, the cells were either left untreated (normoxia) or exposed to 100 µM CoCl_2_ (hypoxia) (Sigma-Aldrich; #C8661). On day 3, the medium was replaced, and cells were either exposed to DMSO (Roth; #A994.1) alone at maximal 0.1% or to compound (**1s**) at the concentrations 2 nM, 5 nM, 10 nM, 20 nM, or 50 nM for 1 h. An amount of 10 ng/mL TNFα was applied as a positive control. Subsequent cell lysis was performed in RIPA-buffer (50 mM tris/HCl (Roth; #9090.2), 1% triton X-100 (Sigma-Aldrich; #X100)/, 0.5% sodiumdeoxycholat (Sigma-Aldrich; #D6750), 0.1% sodium dodecyl sulfate (SDS) (Roth; #CN30.1), 150 mM NaCl (Roth; #3957.1), 1 mM EDTA (Roth; #3053.1), pH 7.5) supplemented with the protease inhibitor Pierce™ (Thermo Fisher Scientific; #88666) and phenylmethanesulfonylfluoride (Sigma-Aldrich; #P7626). An amount of 20 µg of the total protein was separated on a ready-to-use 12% SDS-gel (Bio-Rad, Düsseldorf, Germany; #456-1044). A protein ladder was purchased from (Bio-Rad; #1610374). For IκBα protein detection, the blotting membrane was blocked with 5% BSA (Roth; #8076.2) in a tris buffered saline (TBS; 50 mM tris-HCl (Roth; #9090.2), 150 mM NaCl (Roth; #3957.1), pH 7.4)/ 0.1 % tween^®^-20 (Roth, #9127.1) (TBST); probed with primary antibodies at 4 °C overnight: anti-IκBα (Cell Signaling, Leiden, The Netherlands; #9242; rabbit IgG) and anti-vinculin (Cell Signaling; #13901; rabbit IgG), both at 1:1000 in 1% BSA/TBST; washed four times in TBST; probed with the species appropriate HRP-conjugated secondary antibody: (Thermo Fisher Scientific; #31460; goat-anti-rabbit IgG) at 1:2500 in 1% BSA/TBST; and washed again four times in TBST. For protein detection via chemiluminescence, the membrane was incubated in HRP-substrate (Thermo Fisher Scientific; #34577) for 5 min and the signal was recorded using the Fusion Pulse TS microscope (VILBER, Eberhardzell; Germany). Signal quantification was performed using the software EvolutionCapt (VILBER). For target protein normalization, the protein level of IκBα was normalized to the housekeeping protein vinculin. 

#### 4.8.3. Cell Cycle Studies

To investigate cell cycle progression in the presence of compound (**1s**) or paclitaxel (Santa Cruz Biotechnology; #sc-201439), we quantified the DNA content in the cell. On day 1, the cells were seeded in 1 mL into a 24 well plate (Greiner; #662160). On day 2, MDA-MB-468-UnaG were pre-treated either without (normoxia) or with 100 µM CoCl_2_ (hypoxia) (Sigma-Aldrich; #C8661) for 24 h before compounds were applied. On day 2 for MDA-MB-231 or on day 3 for MDA-MB-468-UnaG, paclitaxel was applied at the concentrations 15 nM, 37 nM, and 100 nM, and compound (**1s**) was applied at concentrations ranging from 2 nM to 1000 nM. Post-treatment cells were washed in 1× PBS (Thermo Fisher Scientific; #14190); fixed in 70% (*v*/*v*) ethanol overnight at 4 °C; washed again in 1× PBS; and, finally, DNA was stained for 15 min at 37 °C in the presence of 10 µg/mL propium iodide (PI; Sigma-Aldrich; #4864) and 100 µg/mL RNase A (Thermo Fisher Scientific; #EN0531) dissolved in 0.1% triton™ X-100 (Sigma-Aldrich; #X100), dissolved in PBS. The fluorescence signal resulting from PI-DNA complex formation was recorded using the flow cytometer CytoFlex (Beckmann Coulter, Krefeld, Germany). The cell cycle phase population was determined based on the DNA content in a set of chromosomes (n) in a cell using the integrated software CytExpert (Beckmann Coulter): sub-G0/G1 (<2n), G0/G1 (2n), S (>2n, <4n), G2/M (4n) or hyperploid cells (>4n) (see Figure S10).

### 4.9. Statistical Analysis

The numeric results are depicted as mean ± standard deviation (SD) or mean ± standard error of the mean (SEM) derived from at least three independent biological replicates (*n* = 3). Non-linear regression curves for the drug dose–response studies, calculation of the half maximal inhibitory concentration (IC_50_), and statistical analysis were performed with GraphPad Prism (Graphpad Software Inc., CA, USA; version 9.3.1). Data displayed in columns or stacked bars were illustrated with GraphPad Prism (Graphpad Software Inc.; version 9.3.1). Statistical difference between the two treatment groups was calculated with an unpaired and two-tailed *t*-test. Statistical difference between more than two treatment groups was calculated with the two-way analysis of variance ANOVA or one-way ANOVA with post analysis using Dunett’s test for comparison of all groups with one another, or Tukey’s test to compare all groups versus one control group. Statistical significance is stated with *p* < 0.05: ns = not significant, * *p* < 0.05; ** *p* < 0.01, *** *p* < 0.001). 

## 5. Conclusions

PAs show a broad range of bioactivities in a variety of cancers. In TNBC we validated dose-dependent anti-inflammatory, anti-hypoxic, anti-migratory, and anti-proliferative activities with an initial SAR for the anti-inflammatory activity showing compound (**1**)/(**1s**), namely *O*-methyltylophorinidine, as the most efficacious compound. 

We describe the class of PAs as a multi-targeting class of natural products with efficacious inhibition of NFκB and HIF, and thus modulating chemoresistance-driving pathways. Although it is already known that PAs are able to address NFκB in various disease settings, we demonstrated that NFκB blockade in TNBC is maintained under hypoxic conditions, presumably by targeting HIF. Inflammation and hypoxia drive tumor progression and are limiting factors for drug sensitivity. Blocking both pathways by PAs might enhance paclitaxel sensitivity in TNBC. Furthermore, PAs maintain the blockade of tumor growth and invasion in a 3D co-culture model while their mode of action may differ from the mode of action of paclitaxel. Further studies need to focus on combinational treatment approaches, and more in-depth SAR studies are inevitable for drug design to prevent neurotoxicity and optimize drug application.

## Figures and Tables

**Table 1:** IC_50_-values determined for NFκB inhibition and reduction of cell viability.

	NFκB Inhibition		Reduction of Cell Viability
**Compound**	**2 h ^1^** **IC_50_ [nM] ^4^**	**24 h ^2^** **IC_50_ [nM] ^4^**	**24 h ^3^** **IC_50_ [nM] ^4,5,6^**
(**1**)	17.1 ± 2.0	3.7 ± 1	13.6 ± 0.4 ^5^
(**2**)	211.8 ± 69.9	38.2 ± 14.2	117.9 ± 35.0 ^4^
(**3**)	284.9 ± 60.4	114.5 ± 17.9	55.3 ± 13.2 ^4^
(**4**)	83.0 ± 14.7	28.3 ± 5.6	127 ± 21.4 ^4^
(**5**)	>1000	>1000	>1000 ^6^
(**6**)	>1000	>1000	>1000 ^6^
**Paclitaxel**	>1000	>1000	37.4 ± 8.6 ^4^

NFκB inhibition was tested after ^1^ 2-h or ^2^ 24-h treatment with the NFκB inhibition assay. ^3^ Cell viability was determined 24 h post-treatment with the 2D cell viability assay. ^4^ Mean ± SD (*n* ≥ 3). ^5^ Mean ± SD (*n* = 2). ^6^ Mean ± SD (*n* = 1).

**Table 2:** IC_50_-values determined for the reduction of cell viability in the 3D co-culture spheroids.

Compound	Reduction of Cell Viability 72 h ^1^ IC_50_ [nM] ^2^
(**1**)	21.7 ± 2.5
(**2**)	441.5 ± 70.8
(**3**)	476.7 ± 160.4
(**4**)	517.7 ± 76.8
(**5**)	>1000
(**6**)	>1000
**Paclitaxel**	43 ± 14.3

^1^ Duration of treatment. ^2^ Mean ± SD (*n* = 3).

**Table 3:** Final compound concentration in the drug dose–response studies.

Compound	Dilution Factor	Concentration Range
(**1), (2), (3), (4), (5), (6**)	4	1000 nM to 0.06 nM
(**1s**)	3	100 nM to 0.05 nM
**Paclitaxel**	3	1000 nM to 0.5 nM

Compounds were applied in triplicates in the NFκB inhibition, 2D and 3D cell viability assay, respectively.

## Data Availability

Not applicable.

## Supplementary Materials

The following supporting information can be downloaded at: https://www.mdpi.com/article/10.3390/ijms231810319/s1.
