# Supplementary material for: Phenanthroindolizidine Alkaloids Isolated from Tylophora ovata as Potent Inhibitors of Inflammation, Spheroid Growth, and Invasion of Triple-Negative Breast Cancer
Source: Int J Mol Sci. 2022 Sep 7;23(18):10319. doi: 10.3390/ijms231810319 (PMC9499467; doi:10.3390/ijms231810319)
Supplement: Supplementary file 1 [file ijms-23-10319-s001.zip › ijms-1841413-supplementary.pdf]

Supplementary Data

1 Supplementary Figures

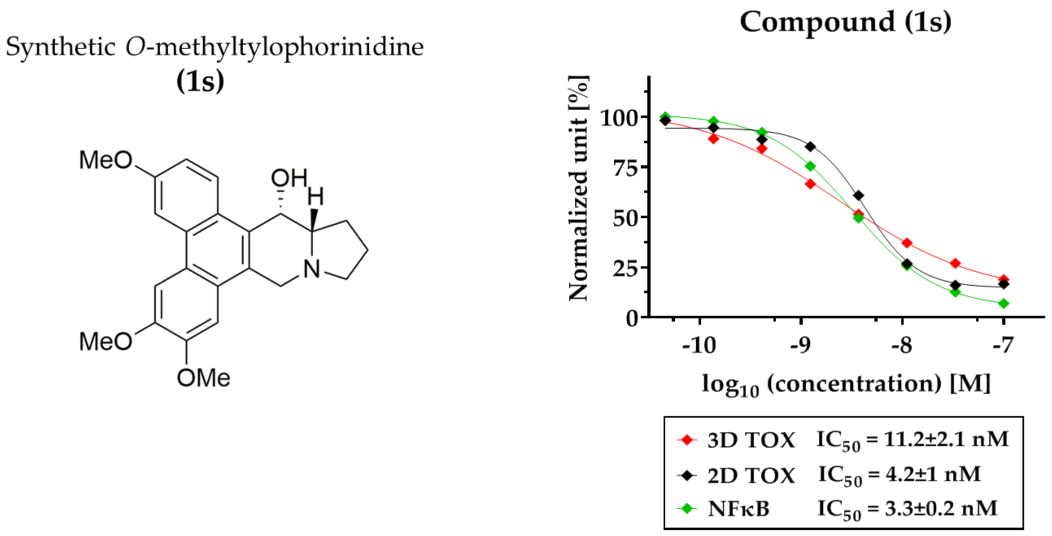

(a) (b)

Figure S1 Bioactivity of the chemically synthesized *O*-methyltylophorinidine (**1s**). (**a**) Molecule structure of (**1s**) is illustrated using ChemDraw (PerkinElmer Informatics, Germany; Version: 18.1.0.458). (**b**) Compound (**1s**) was tested in MDA-MB-231 and 3D co-culture in a concentration range starting at 100 nM in a three-fold serial dilution: (**NFκB**) According to the NFκB-inhibition assay, compound (**1s**) was tested within 2 h in NFκB MDA MB 231-nanoluc cells. NFκB-dependent luciferase activity was measured in relative light units and was normalized to the lowest concentration applied (6×10^{-11} M; 100%) (normalized unit). Data points represent the mean ($n = 2$); (**2D TOX**) According to the 2D cell viability assay compound (**1s**) was tested within 24 h in MDA-MB-231 cells. Cell viability was measured in relative fluorescence units and was normalized to the lowest (0%) and highest (100%) value (normalized unit). Data points represent the mean ($n = 3$); (**3D TOX**) According to the 3D cell viability assay compound (**1s**) was tested within 72 h in 3D co-culture spheroids. Cell viability was measured as relative light units and was normalized to the lowest (0%) and highest (100%) value (normalized unit). Data points represent the mean ($n = 3$).

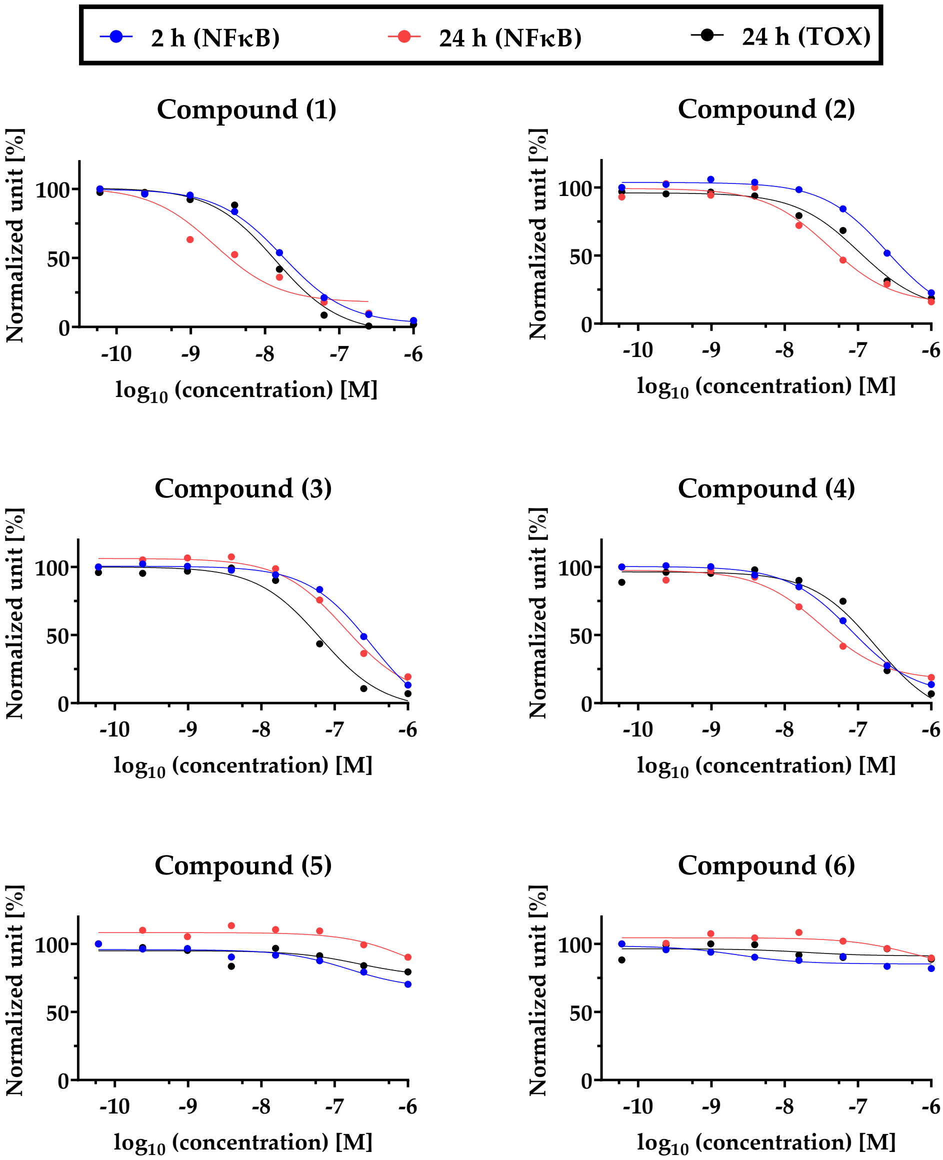

Figure S2 Time- and dose- dependent NFκB-blockade in MDA-MB-231. According to the NFκB inhibition assay, the NFκB-dependent luciferase activity within 2 h or 24 h compound stimulation was recorded as relative light units (RLU). In each individual experiment, the RLU was normalized to the lowest concentration applied (6×10^{-11} M; 100%) (normalized unit). According to the 2D cell viability assay, cell viability was determined within 24 h compound stimulation as relative fluorescence units (RFU). of the cytotoxic staurosporine as control . In each individual experiment, RFU was normalized to the lowest (staurosporine, 0%) and highest (untreated, 100%) RFU value (normalized unit). Data points represent the mean±SD ($n = 3$).

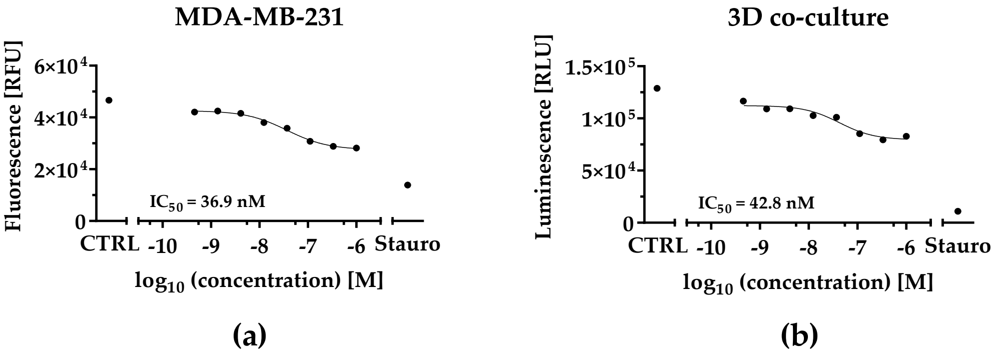

Figure S3 Representative dose-response inhibition by paclitaxel in monolayer cells and 3D co-culture spheroids**.** **(a)** MDA-MB-231 were grown for 24 h in the presence of paclitaxel. Cell viability-dependent fluorescence was determined post-treatment according to the 2D cell viabilityassay**.** **(b)** The co-culture spheroids were grown for 72 h in the presence of paclitaxel. Cell viability-dependent luminescence was determined post-treatment according to the 3D cell viability method**.** **(a,b)** Untreated cells and spheroids (CTRL) or treatment with 20 µM of cytotoxic staurosporine (Stauro) served as control. Data points represent one individual experiment.

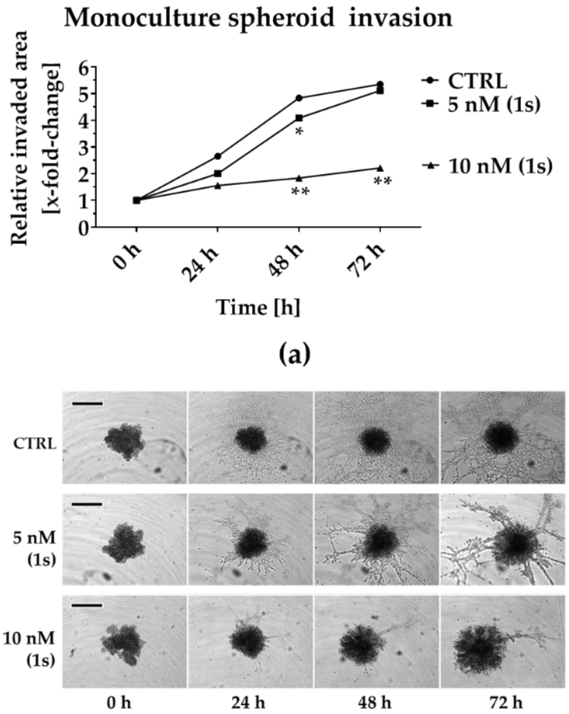

with manually picked positions (white squares) to determine the outline (yellow line) of the spheroid area within 0 h and after 48 h treatment. (**b**) Representative brightfield images of the monoculture spheroids. Scale bar: 500 µm. (**c**) Invaded area is calculated by normalizing the spheroid area after 48 h treatment to the spheroid size at 0 h (which was set to 1; x-fold-change

Figure S4 Compound (**1s**) blocks TNBC spheroid invasion into a matrigel®-based extracellular matrix in a dose-dependent manner. Monoculture spheroids composed of MDA-MB-231 were generated for 72 h before matrigel® was applied for matrix formation. Spheroids were grown for another 72 h without (CTRL) or in the presence of 5 nM or 10 nM compound (**1s**). (**a**) Invaded area is normalized to the spheroid area at 0 h (which was set to 1; x-fold-change). Statistical difference was calculated using one-way-ANOVA analysis with performing Tukey's Multiple Comparison Test

with depicting the results within each time point separately vs. CTRL (* $p \leq 0.05$, ** $p \leq 0.01$). Data points represent the mean ($n = 3$). (**b**) Representative spheroids. Scale bar: 500 µm.

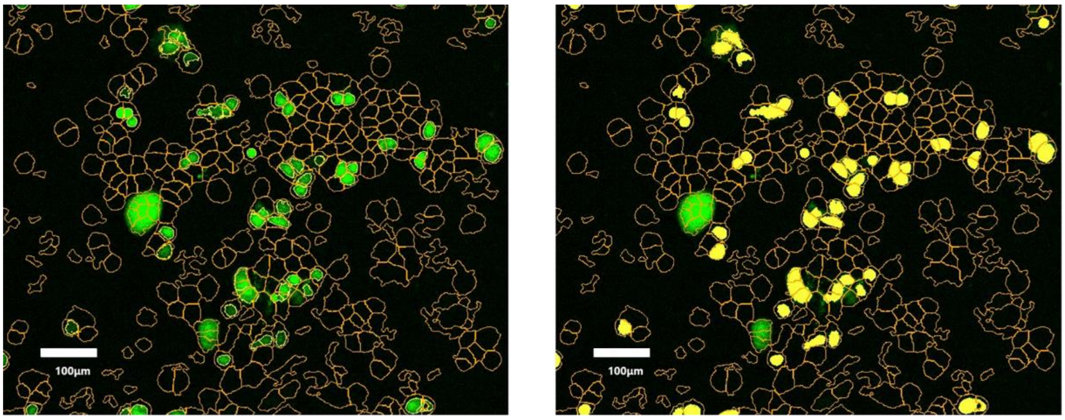

(a) (b)

Figure S5 Quantification of hypoxia via the reporter gene UnaG in MDA-MB-468-UnaG. Fluorescence images were recorded using the spinning-disk CQ1 microscope at 100× magnification. **(a, b)**Cells, ′recorded using brightfield, were recognized by a machine learning algorithm (yellow outlines). **(a)** HIF-dependent UnaG expression (green area) was recorded using the 488 nm channel. Despite cell clone selection of HIF-mediated UnaG expressing cells, UnaG is not expressed in all cells. Thus, UnaG expressing cells are recognized within a particular cell size (yellow area). Fluorescence intensity of UnaG was measured within the UnaG expressing cells. Scale bar: 100 µm.

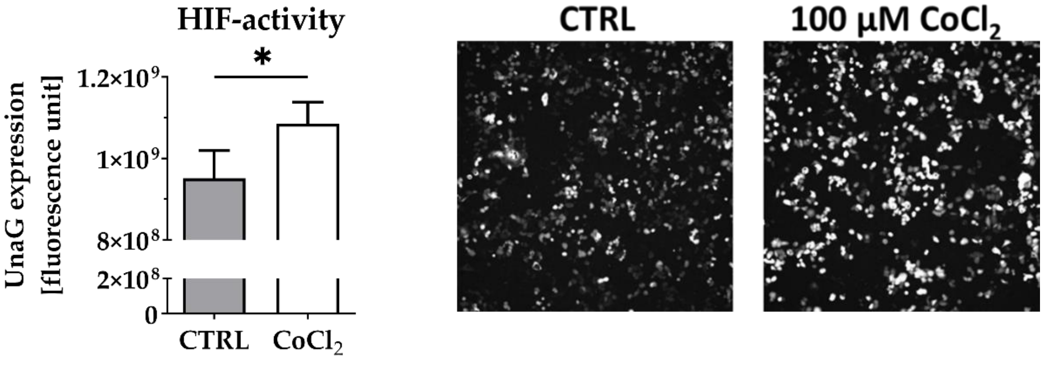

(a) (b)

Figure S6 $CoCl_2$ simulates hypoxia by stimulating HIF signaling. MDA-MB-468 cells were grown without (CTRL) or in the presence of 100 µM $CoCl_2$ for 24 h. (**a**) Fluorescence intensity of HIF-mediated UnaG expression was determined in UnaG-expressing cell clones. Statistical difference was calculated with paired t-test (* $p < 0.05$). Columns represent the mean ± SEM ($n = 3$). (**b**) Representative images for UnaG fluorescence (white area) recorded at 100× magnification.

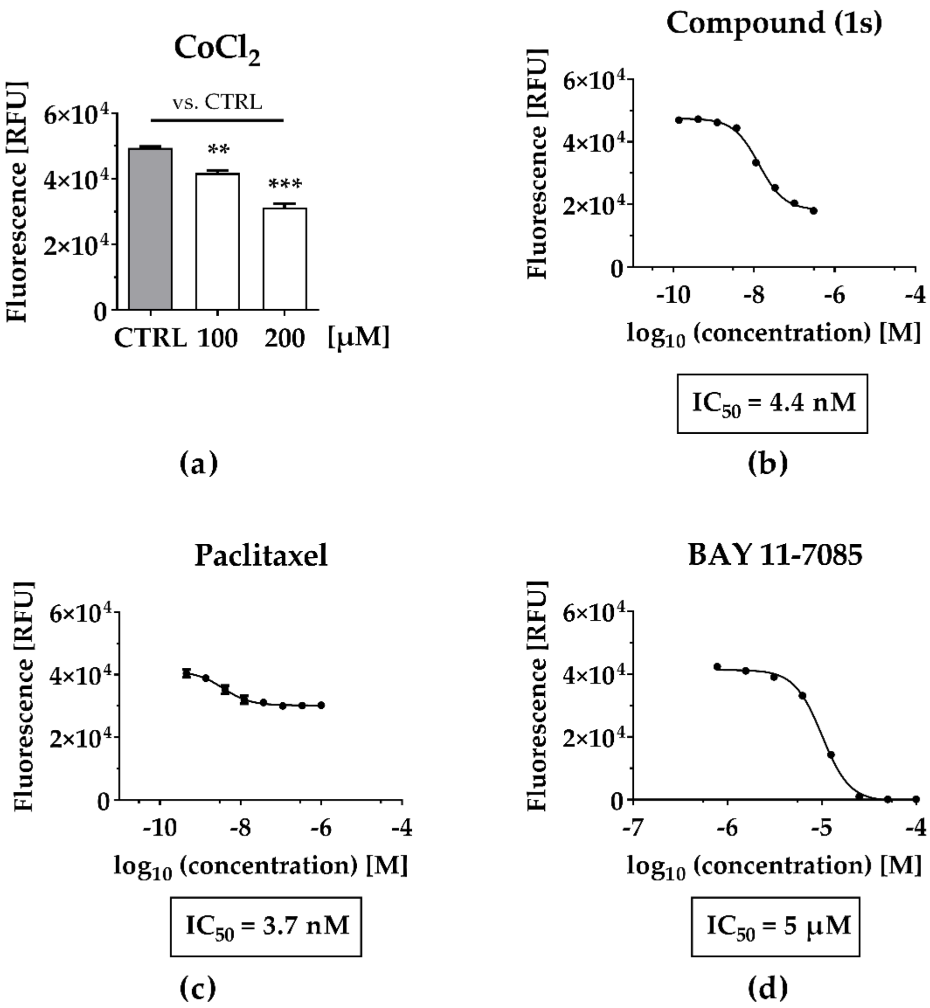

Figure S7 Dose-response inhibition for $CoCl_2$, compound (**1s**), paclitaxel and BAY 11-7085 in MDA-MB-468-UnaG. 2D cell viability was determined within 24 h in untreated cells (CTRL) or in cells exposed to **(a)** $CoCl_2$, **(b)** compound **(1s)**, **(c)** paclitaxel or **(d)** BAY 11-7085. Compound concentration was applied in triplicates in a three-fold serial dilution starting at 100 nM for (**1s**), at 1000 nM for pacliaxel and at 50 μM for BAY 11-7085. (**a**) Columns or **(b-d)** data points represent one independent experiment. **(a)** Statistical difference was calculated using two-way-ANOVA analysis vs. CTRL (** $p < 0.01$, *** $p < 0.001$).

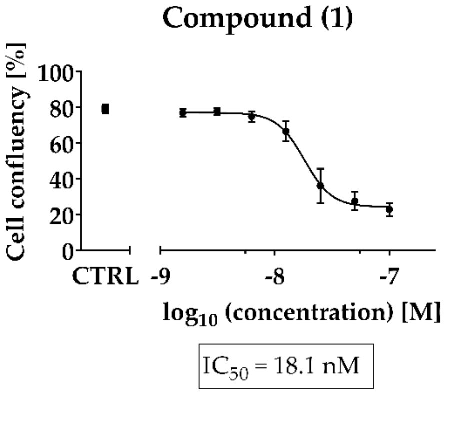

Figure S8 Compound (**1**) blocks proliferation within 48 h in a dose-dependent manner in MDA-MB-231. Cells were grown for 48 h without (CTRL) or in the presence of compound (**1**). Proliferation based on cell confluency was determined within 48 h as previously described [23]. Data points represent the mean (n = 3).

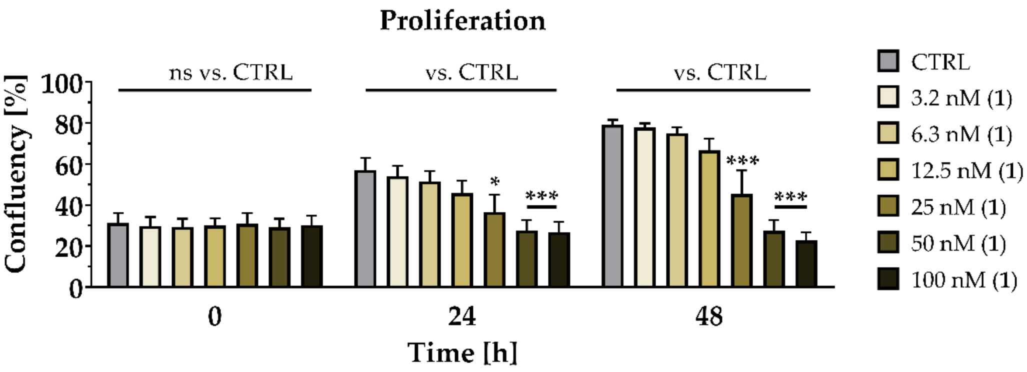

Figure S9 Compound (**1**) blocks proliferation in a dose-and time-dependent manner in MDA-MB-231. Cell proliferation based on cell confluency was determined within 48 h as previously described [23] starting with 1 × 10^4 cells per well. Columns represent the mean ± SEM (n = 3). At each time point, statistical significance in proliferation was calculated with two-way ANOVA analysis vs. CTRL (ns = not significant, * $p < 0.05$, *** $p < 0.001$).

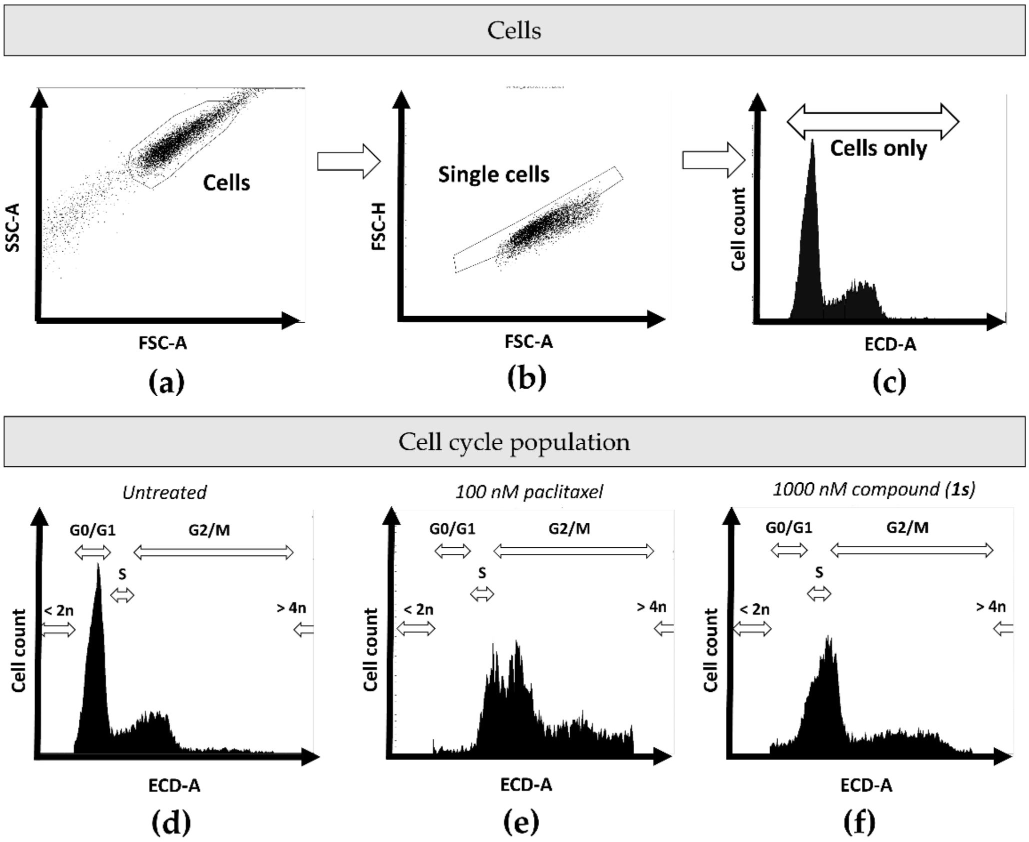

Figure S10 Quantification of cell cycle phase population using CytExpert. Cells were exposed maximal 72 h to paclitaxel or incubated for maximal 72 h in the presence of compounds. Signal of the PI-DNA complex was recorded within the ECD-A channel using the flow cytometer CytoFlex. (**a**) Cells were identified in the recorded events by gating for the forward (FSC-A) and sideward scatter area (SSC-A). (**c**) Single cells were identified and subsequently gated for **(b)** single cells using the

FSC-A and FSC-height (H). **(c)** To evaluate cell cycles phases, the range of analyzed cells was determined within the **(b)** single cells by excluding recorded events that extend the proportional distribution of the DNA chromosome set (n): < 2n; > 4n. **(d-f)** Gating for the cell cycle population sub-G0/G1 (< 2n), G0/G1 (2n), S, G2/M (4n) or hyperploid (> 4n) cells was done manually and maintained for each individual experiment and each individual time point of treatment. The cell phase population G0/G1 was determined in relation to the **(c)** untreated group and G2/M was determined in relation to the highest concentration applied for **(d)** paclitaxel and **(e)** compound (**1s**). Histograms were illustrated with CytExptert.

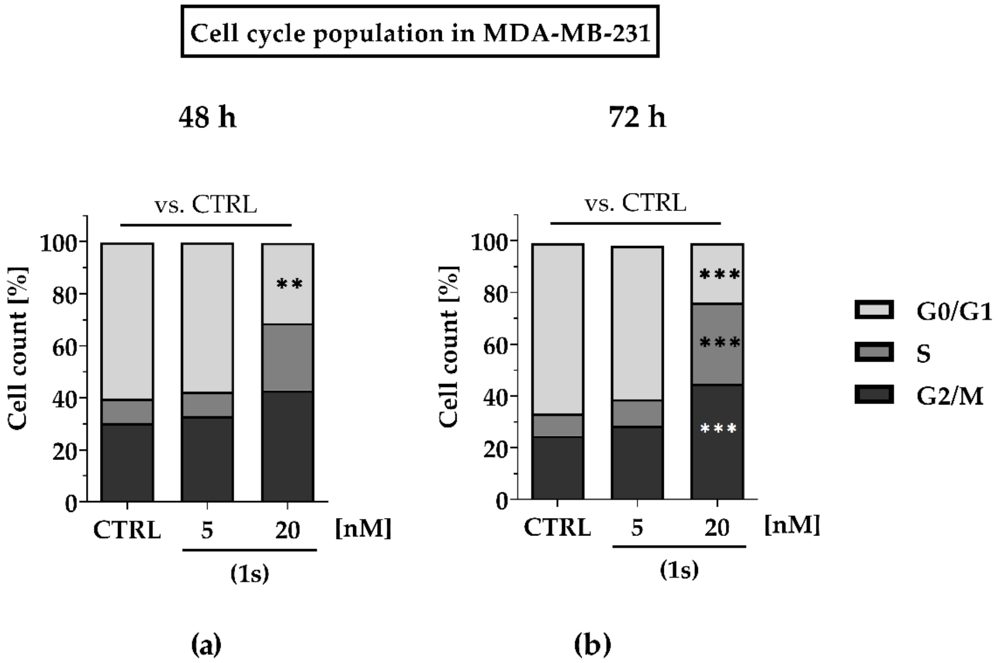

Figure S11 Compound (**1s**) enhances cell cycle population at the S-and G2/M phase in MDA-MB-231 cells in a dose-and time-dependent manner. Cells were incubated for **(a)** 48 h or **(b)** 72 h without (CTRL) or in the presence of 5 nM and 20 nM compound **(1s)**. Cell cycle phase population (G0/1; S; G2/M) was determined based on the signal of the PI-DNA complex using CytExpert. **(a)** Representative cell cycle distribution upon paclitaxel treatment for MDA-MB-231 and MDA-MB-468-UnaG cells. Statistical difference was calculated using two-way-ANOVA analysis vs. CTRL (** $p < 0.01$, *** $p < 0.001$). Stacked bars represent the mean ($n = 3$).

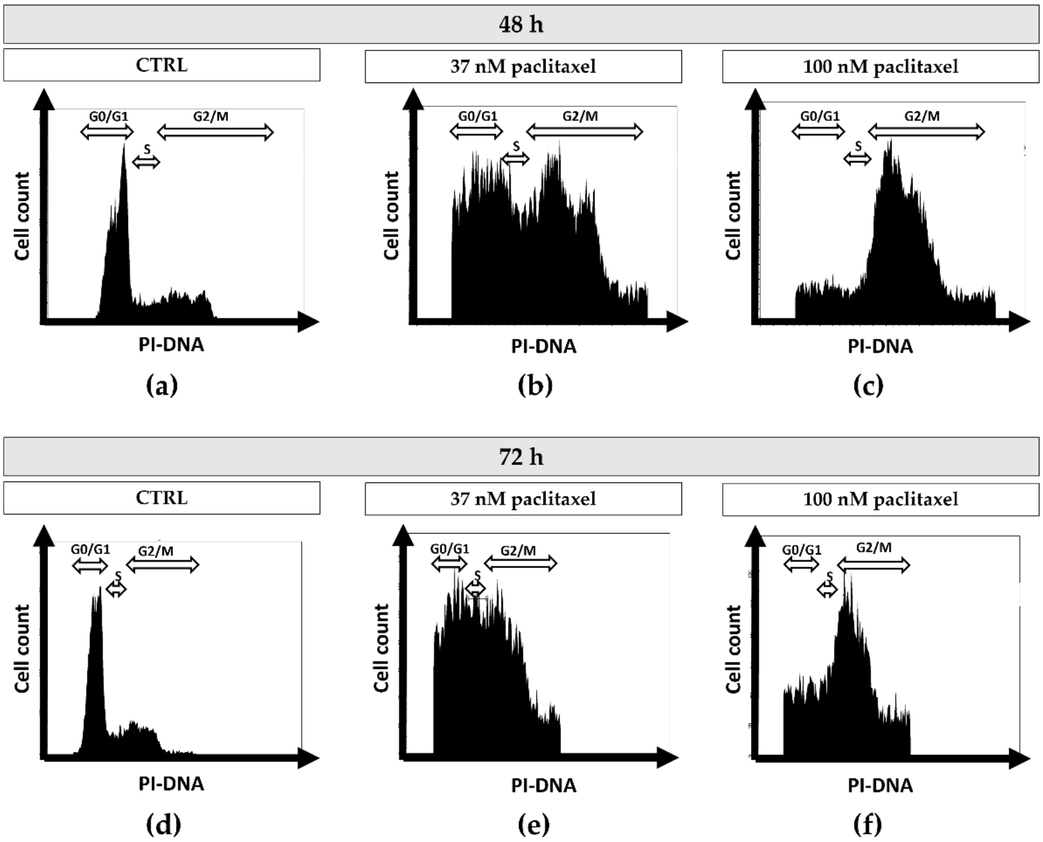

Figure S12 Paclitaxel arrests cell cycle at the G2/M phase in MDA-MB-468-UnaG. Cells were grown for **(a–c)** 48 h or **(d–f)** 72 h without (CTRL) or in the presence of paclitaxel. Cell cycle phase population (G0/1; S; G2/M) was determined based on the signal of the PI-DNA complex using CytExpert. Representative histograms for G2/M phase arrest in MDA-MB-231 and MDA-MB-468-UnaG depicting the cell count within the PI-DNA signal were illustrated with CytExpert.

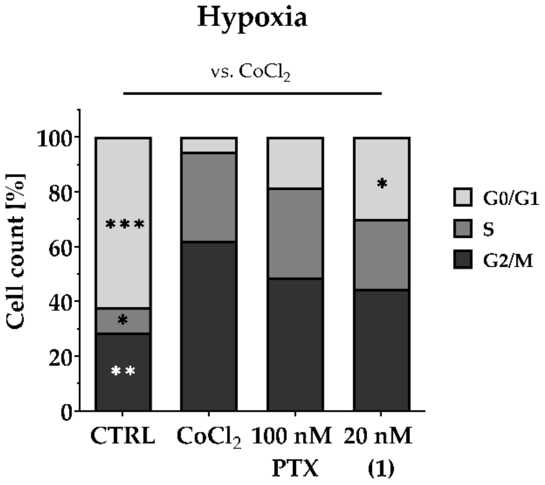

Figure S13 $CoCl_2$-simulated hypoxia arrests cell cycle at the G2/M phase in MDA-MB-468-UnaG. For hypoxia simulation, cells were pre-treated with 100 μM $CoCl_2$ for 24h. Cells were untreated (CTRL) or stimulated with paclitaxel (PTX) or compound (**1s**). Cell cycle phase population (G0/1; S; G2/M) was determined based on the signal of the PI-DNA complex using CytExpert. Stacked bars represent the mean ($n = 3$). Statistical difference was calculated using two-way-ANOVA analysis vs. $CoCl_2$ (* $p = 0.05$, *** $p < 0.001$).

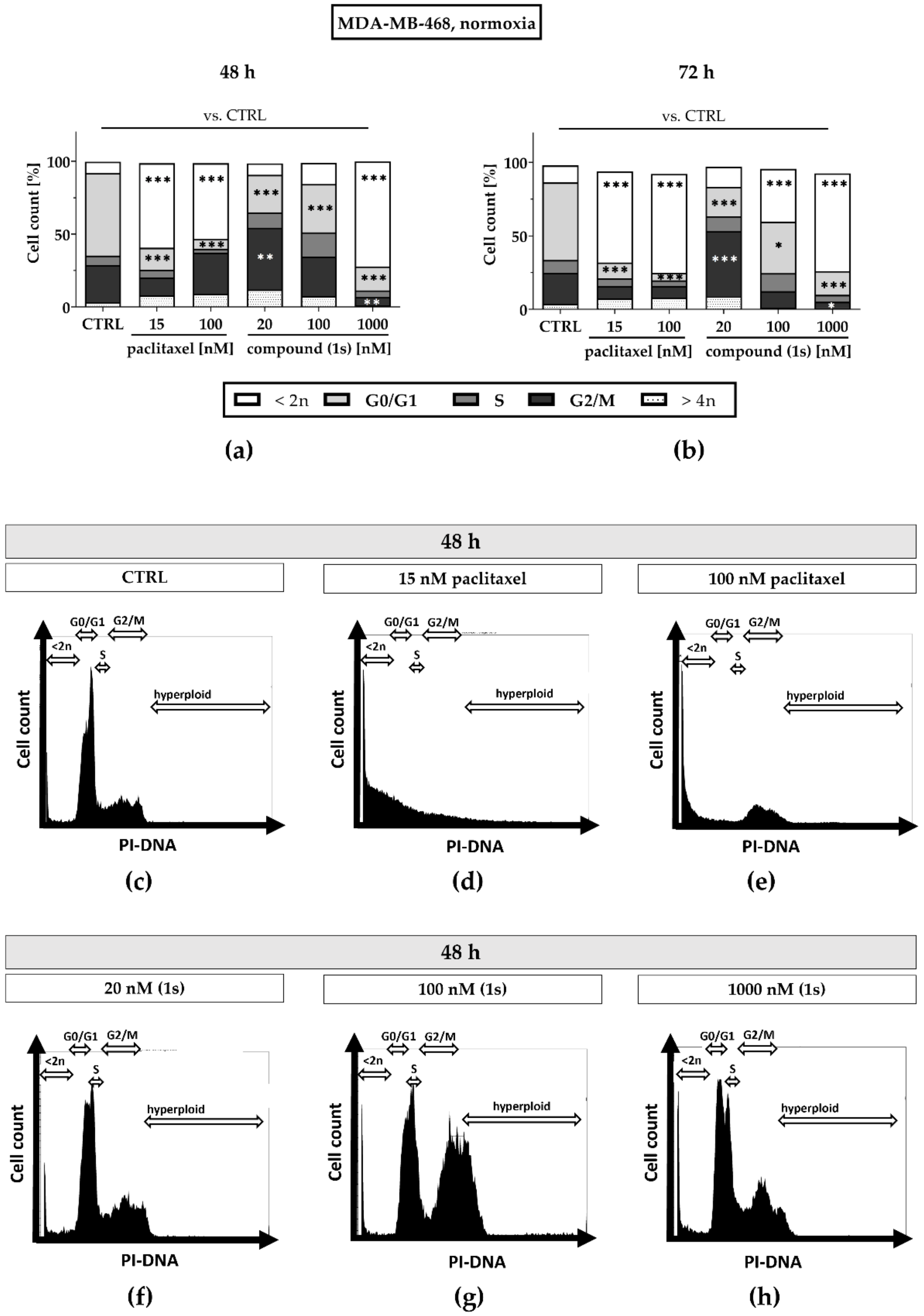

Figure S14 Paclitaxel and compound (**1s**) induce apoptosis. Cells were grown for 48 h **(a, b, c, f)** without (CTRL) or in the presence of **(a, b, d,e)** paclitaxel or (**a,b,g,h**) compound (**1s**). Cell cycle phase population (sub-G0/G1 (<2n); G0/1; S; G2/M; hyperploid cells (> 4n)) was determined based on the signal of the PI-DNA complex using CytExpert. **(a, b)** Stacked bars represent the mean ($n = 3$). Statistical difference was calculated using two-way-ANOVA analysis vs. CTRL (* $p < 0.05$, *** $p < 0.001$). **(c–h)** Representative histograms depicting the cell count within the PI-DNA signal were illustrated with CytExpert.

2 Supplementary Tables

Table S1 IC_{50}-values for reduction of cell viability determined in CAFs.

**Compound**	**Reduction of Cell Viability** [1] **24 h** [2] **IC_{50} [nM]** [3]
(**1**)	2.7 ± 0.9
(**2**)	19.7 ± 3.7
(**3**)	26.7 ± 3.1
(**4**)	33.5 ± 12.7
(**5**)	> 1000
(**6**)	> 1000

[1] Cell viability of CAFs was determined 24 h post-treatment with the 2D cell viability assay. CAFs were seeded at a cell density of 2.5×10^3 cells per well. Compound concentration was applied in triplicates in a four-fold serial dilution starting at 1000 nM. [2] Duration of treatment. [3] Mean ± SD (n = 3).

Table S2 IC50-values determined for BAY 11-7085 in NFκB inhibition and cell viability.

**Compound**	**2 h** [1] **IC_{50} [nM]**	**24 h** [2] **IC_{50} [nM]**	**24 h** [3] **IC_{50} [nM]**
**BAY 11-7085**	5500 ± 2000[4]	12,623 ± 1542[4]	12,500 ± 1450[5]

NFκB-inhibition was tested after [1] 2 h or [2] 24 h treatment with the NFκB-inhibition assay. [3] Cytotoxicity was determined 24 h post-treatment with the 2D cell viability assay. Final compound concentration was applied in triplicates in a two-fold or three-fold serial dilution starting at [1,3] 100 μM or [2] 50 μM. [4] Mean ± SD (*n* = 3). [5] Mean ± SD (*n* = 2).
